# Functional network reorganization after magnetic resonance-guided focused ultrasound thalamotomy in tremor-dominant Parkinson's disease patients

**DOI:** 10.1016/j.nicl.2026.104058

**Published:** 2026-09-05

**Authors:** Jinlong Liu, Veronika Purrer, Jonas Krauss, Valeri Borger, Markus Essler, Alexander Radbruch, Ullrich Wüllner, Neeraj Upadhyay, Henning Boecker

**Affiliations:** a University of Bonn, University Hospital Bonn, Clinical Functional Imaging Group, Department of Nuclear Medicine, Venusberg-Campus 1, 53127, Bonn, Germany; b German Center for Neurodegenerative Diseases (DZNE), Venusberg-Campus 1, 53127, Bonn, Germany; c University of Bonn, University Hospital Bonn, Clinic for Parkinson's Disease, Sleep Disorders, and Movement Disorders, Venusberg-Campus 1, 53127, Bonn, Germany; d University of Bonn, University Hospital Bonn, Department of Neurosurgery, Venusberg-Campus 1, 53127, Bonn, Germany; e University of Bonn, University Hospital Bonn, Department of Nuclear Medicine, Venusberg-Campus 1, 53127, Bonn, Germany; f University of Bonn, University Hospital Bonn, Department of Neuroradiology, Venusberg-Campus 1, 53127, Bonn, Germany

**Keywords:** Parkinson's disease, MRgFUS, Brain networks, Graph theory analysis, Small-worldness

## Abstract

**Background:**

Magnetic resonance-guided focused ultrasound (MRgFUS) thalamotomy alleviates tremor in tremor-dominant Parkinson's disease (TD-PD). However, its effects on the functional network topology remain unclear.

**Methods:**

Twenty-one TD-PD patients underwent unilateral MRgFUS thalamotomy and clinical and MRI assessments. For the overall cohort, graph theory analysis was employed to examine differences in small-world properties and modular structure between baseline and 6 months after MRgFUS. Changes in topological properties were assessed using paired *t*-tests or Wilcoxon tests, and their associations with clinical improvement. Additionally, subgroup analyses were performed on responder (*n* = 10) and relapser (*n* = 9) data.

**Results:**

Tremor scores decreased significantly after MRgFUS contralateral to the lesioned thalamus. Significant increases were observed in small-worldness and normalized clustering coefficient after MRgFUS. Inter-modular connectivity showed trend-level enhancement between cortical-basal ganglia and thalamic modules, and between cerebellar and temporal-hippocampal modules. In responder, significant increases were observed in small-worldness, normalized clustering coefficient, and global efficiency, whereas characteristic path length showed a significant decrease. Changes in global efficiency and characteristic path length were significantly correlated with clinical improvement.

**Conclusion:**

MRgFUS thalamotomy for TD-PD induced partial reorganization of small-world properties, indicating a shift toward a more integrated network topology, especially in responder. Changes correlated with tremor reduction and improved functional disability, highlighting that effective treatment impacts on global network integration.

## Highlights

• MRgFUS altered functional network topology in TD-PD.
• Small-worldness and relative clustering increased after MRgFUS.
• Responder showed enhanced global integration.
• Changes in global efficiency and characteristic path length correlated with clinical improvement.

## 1 Introduction

Parkinson's disease (PD) is a heterogeneous neurodegenerative disorder characterized by a wide spectrum of motor manifestations, among which tremor represents a prominent feature (Abusrair et al., 2022). Tremor is frequently insufficiently controlled by pharmacological therapies, resulting in substantial functional impairment and reduced quality of life (Pasquini et al., 2023). Magnetic resonance-guided focused ultrasound (MRgFUS) thalamotomy has emerged as a non-invasive ablative treatment and demonstrated clinical efficacy in reducing medication-refractory tremor (Bond et al., 2017; Purrer et al., 2022). Clinical outcomes following MRgFUS thalamotomy in tremor-dominant Parkinson's disease (TD-PD) are heterogeneous. Although many patients experience sustained tremor relief, approximately 30–50% exhibit only transient benefit, with tremor recurrence occurring within weeks after treatment (Tian et al., 2023; Peters et al., 2024; Braccia et al., 2025). Previous studies have categorized TD-PD patients as ‘responder’ or ‘relapser’ based on post-operative tremor improvement (Braccia et al., 2025; Peters et al., 2025).

Tremor generation in PD is commonly conceptualized as a network-level dysfunction. Neuroimaging studies suggest that parkinsonian tremor involves abnormal interactions between basal ganglia and cerebello-thalamo-cortical (CTC) circuitry, which is embedded in the dimmer-switch model (Helmich et al., 2012; Dirkx and Bologna, 2022). According to this framework, the basal ganglia trigger tremor episodes, while the CTC circuitry determines tremor amplitude (Helmich et al., 2012; Dirkx and Bologna, 2022). Accumulating evidence suggests that MRgFUS can modulate aberrant brain circuits at a network level. Resting-state functional MRI (rs-fMRI) has shown that MRgFUS thalamotomy alters connectivity within tremor-related networks, with changes correlating with clinical improvement (Stanziano et al., 2021). Network-based statistics analyses have identified an MRgFUS-sensitive subnetwork reflective of hand tremor recovery and surgical process (Lin et al., 2021). Dahmani et al. (Dahmani et al., 2023) demonstrated that MRgFUS thalamotomy induced tremor improvement associated with functional reorganization between the brain's hand region and the cerebellum.

Graph theory analysis provides a framework for characterizing brain network topology (Sporns, 2018). Small-world properties describe an optimal balance between functional segregation and integration that supports efficient information transfer, whereas modularity reflects the extent to which networks are organized into relatively independent communities (Rubinov and Sporns, 2010). Disruptions of small-world properties have been reported in PD, typically characterized by reduced clustering coefficient and increased characteristic path length, together with a breakdown in functional modular organization (Prasad et al., 2024; Suo et al., 2022; Tinaz et al., 2017).

However, little is known about the effects of MRgFUS on functional network topology in PD. Disrupting tremor-generating mechanisms at the level of the thalamic ventral intermediate nucleus (VIM) by MRgFUS allows to investigate the impact of this novel treatment on functional network reorganization, both within and beyond the CTC circuitry and the connected basal ganglia networks. As of yet, the topological network changes associated with MRgFUS in TD-PD – and how these relate to differential clinical outcomes including the phenomenon of tremor relapse – have not yet been fully elucidated.

Given that disruptions of small-world properties and functional modular organization have been reported in PD (Prasad et al., 2024; Suo et al., 2022; Tinaz et al., 2017), and assuming that rhythmic oscillations related to tremor may disrupt the efficient flow of information in TD-PD, we hypothesize that interrupting the related activity in the VIM using MRgFUS could facilitate the redistribution of information flow across affected brain networks. MRgFUS may therefore result in a more efficient and integrated configuration of functional brain networks, characterized by improvements in small-world properties, increased global efficiency and a reorganized modular structure. Consequently, we hypothesize that these network-level changes will be more pronounced in responder than in relapser to MRgFUS thalamotomy.

## 2 Materials and methods

### 2.1 Patients

Patients diagnosed with TD-PD (Stebbins et al., 2013) underwent unilateral VIM-MRgFUS treatment (ExAblate Neuro 4000, Insightec) between 2019 and 2024 at University Hospital Bonn. Detailed inclusion and exclusion criteria for MRgFUS treatment can be found in the German Clinical Trials Registry (DRKS00016695). Ethical approval was obtained from the local ethics committee of the University Hospital Bonn (No. 314/18), and the study was carried out in accordance with the Declaration of Helsinki.

### 2.2 Clinical assessment

Tremor severity was assessed using the Fahn-Tolosa-Marin Clinical Rating Scale for Tremor (FTM) (Fahn et al., 1988) and tremor-related items (3.15–3.18) from the Movement Disorder Society-Unified Parkinson's Disease Rating Scale (MDS-UPDRS) part III (Goetz et al., 2008) at baseline, 3 days and 6 months after MRgFUS, with all evaluations performed in the medication “off” state (in the out-patient condition at least 4 h after the last intake of antiparkinsonian drugs). The FTM scale consists of three subscales. FTM A and B assess tremor severity and were combined to generate a modified FTM A/B score informing on tremor specific qualities and calculated separately for the treated and untreated sides. FTM C measures tremor-related functional disability in activities of daily living (e.g., eating, drinking, washing, etc.). Tremor improvement on the treated side after MRgFUS was calculated using the following formula: (tremor score at baseline − tremor score at follow-up)/tremor score at baseline × 100%. Patients were categorized as responder (tremor improvement ≥50% at both 3-day and 6-month follow-up) or relapser (tremor improvement ≥50% at 3-day follow-up but <50% at 6-month follow-up), according to previously published classification schemes (Peters et al., 2025; Braccia et al., 2025). Responder classification was primarily based on the tremor improvement in the MDS-UPDRS III, as the MDS-UPDRS III is the standard clinical scale for assessing motor symptoms in PD. For patients without available MDS-UPDRS III tremor scores, the FTM A/B was used for responder classification.

### 2.3 MRgFUS intervention

Before the intervention, all patients underwent a CT scan to ensure a skull density ratio (SDR) of ≥0.3. The treatment target was determined using standard atlas-based stereotactic approximations according to a published procedure (Keil et al., 2020). Diffusion tensor imaging (DTI) was employed to visualize the dentato-thalamo-cortical tract (DTCT) while avoiding the corticospinal tract and medial lemniscus. Initial subthreshold sonications (< 50 °C) were performed to identify the optimal target and exclude potential adverse effects. The detailed procedure was described previously (Keil et al., 2020; Purrer et al., 2022).

### 2.4 MRI acquisition

MRI data were obtained at the University Hospital Bonn at baseline, 3-day and 6-month follow-up. Structural images were acquired using a three-dimensional (3D) T1-weighted Magnetization Prepared-Rapid Gradient Echo (MPRAGE) sequence with the following acquisition parameters: voxel size: 1 × 1 × 1 mm^3^; matrix size: 256 × 256 mm; repetition time (TR): 7.293 ms; echo time (TE): 3.93 ms; flip angle (FA): 15°; scan duration: 4:40 min. The acquisition parameters of rs-fMRI images were as follows: voxel size: 3.59 × 3.59× 3.59 mm^3^; matrix size: 64 × 64 mm; TR: 2.595 s; TE: 5.5001 ms; slice order: interleaved (odd slices first, then even slices); FA: 90°. Each run consisted of 250 rs-fMRI volumes with a total acquisition time of 11 min. During rs-fMRI, participants were asked to lie quietly with eyes closed, remain alert, and refrain from engaging in any directed cognitive activity.

### 2.5 MRI preprocessing

MRI data were preprocessed using CONN 22a (http://www.nitrc.org/projects/conn) based on SPM12 (https://www.fil.ion.ucl.ac.uk/spm/software/spm12/). To ensure hemispheric consistency, data from patients with right VIM treatment were flipped along the right-left axis. The preprocessing steps included: (1) realignment with fieldmap-based distortion correction using SPM's realign & unwarp; (2) slice-timing correction for interleaved acquisition; (3) outlier detection using ART based on global BOLD signal change >5 standard deviations or framewise displacement >0.9 mm (Nieto-Castanon, 2020); (4) functional and anatomical images underwent coregistration and normalization to MNI space, followed by tissue segmentation into gray matter, white matter, and cerebrospinal fluid (CSF), and were subsequently resampled to 2 mm isotropic voxels; (5) spatial smoothing using a 6 mm full width half maximum (FWHM) Gaussian kernel; (6) functional data were denoised by regressing potential confounding effects from white matter (5 CompCor components), CSF (5 CompCor components), motion parameters and their first-order derivatives (12 factors), outlier scans, and linear trends, followed by high-pass filtering of the BOLD timeseries above 0.01 Hz.

### 2.6 Network construction

Functional connectivity matrices were constructed using the Human Brainnetome Atlas (https://atlas.brainnetome.org/), with the atlas resampled to 2 mm isotropic resolution, parcellating the brain into 273 regions of interest (ROIs) (246 cerebral and 27 cerebellar). Mean BOLD time series were extracted for each ROI by averaging all voxel signals within the region, and functional connectivity matrices were generated using Pearson's correlations between all ROI pairs. Correlation coefficients were subsequently converted to z values using Fisher's r-to-z transformation to improve normality. Due to the controversial interpretation of negative correlations in functional connectivity (Anderson et al., 2011; Chang and Glover, 2009), all negative connectivity values were thresholded to 0.

To reduce bias associated with single-threshold selection, brain networks were constructed across a sparsity range of 0.05–0.50 (step = 0.01). To ensure that this range did not yield disconnected networks or violate small-world properties at either end, all participants were required to meet two prerequisites across thresholds: (a) a mean network degree greater than 2 × log(N), where N represents the number of nodes, and (b) preserved small-worldness (σ > 1.1) (Zhang et al., 2011). At each sparsity level, weighted matrices were transformed into binary matrices, with supra-threshold connections set to 1 and sub-threshold connections set to 0.

### 2.7 Network analysis

Graph theory analysis was carried out using the Graph Theoretical Network Analysis toolbox (GRETNA) (https://www.nitrc.org/projects/gretna), with both global and local network metrics computed across all thresholds. Global metrics included small-worldness (σ), characteristic path length (Lp), normalized characteristic path length (λ), clustering coefficient (Cp), normalized clustering coefficient (γ), local efficiency (Eloc), global efficiency (Eg), and modularity (Q). Local metrics included betweenness centrality, degree centrality, and nodal efficiency. Comprehensive explanations of the calculation and interpretation of these metrics are available in prior literature (Rubinov and Sporns, 2010). For each network metric, the area under the curve (AUC) was computed across the predefined sparsity range to provide a scalar summary of network metrics while reducing sensitivity to the choice of a specific threshold (Zhang et al., 2011).

Modular analysis was performed to investigate the community structure of functional brain networks. To obtain a stable and representative modular architecture at the population level, a group-level functional brain matrix was constructed by averaging individual functional connectivity matrices at baseline. The group-mean matrix was thresholded at a sparsity level of 0.15, representing the lowest network density while minimizing spurious connections (Bouyssi-Kobar et al., 2019). To assess the robustness of the identified modular organization, the modular decomposition was additionally repeated at sparsity thresholds of 0.13 and 0.17. Modular structure was identified using a modified greedy optimization algorithm implemented in the Brain Connectivity Toolbox (Rubinov and Sporns, 2010). Based on the detected modules, inter- and intra-modular connections were calculated to characterize connectivity patterns within and between modules. To further explore potential contributors to altered modular interactions, nodal topological measures, including participation coefficient, degree centrality, betweenness centrality, and nodal efficiency, were additionally computed. As a complementary analysis, the functional modular partition was applied to structural matrices to calculate inter- and intra-modular connectivity strength. Detailed preprocessing, structural network construction, and analysis procedures are provided in the Supplementary Methods.

### 2.8 Statistical analysis

Statistical analyses were performed using SPSS 27 (IBM Corp., Armonk, NY, USA). Data normality was assessed using the Shapiro-Wilk test. For the entire cohort, longitudinal changes in network metrics from baseline to six months following MRgFUS were evaluated using paired *t*-tests or Wilcoxon signed-rank tests, depending on data normality. Spearman's rank correlation was applied to evaluate the association between network topological properties and FTM improvement. Based on our hypothesis that MRgFUS leads to a more efficient and integrated functional brain network configuration, one-tailed statistical tests were applied for global network metrics, whereas two-tailed tests were used for all other comparisons. Multiple comparisons were controlled using false discovery rate (FDR) correction for local metrics and modular interactions. Global network metrics were analyzed without multiple-comparison correction (Collantoni et al., 2019; Li et al., 2025). A *p*-value <0.05 was considered statistically significant.

To evaluate the robustness of the primary longitudinal findings, leave-one-subject-out (LOSO) analyses were performed by repeating the longitudinal analyses while iteratively excluding one participant at a time. To assess the potential influence of head motion on network metrics, Spearman's rank correlation analyses were performed between the longitudinal changes in significant network metrics and individual mean framewise displacement (FD).

Subsequently, patients were classified into responder and relapser groups based on predefined clinical criteria. Within each subgroup, longitudinal changes in network metrics between baseline and post-treatment were assessed following the same procedures as for the overall cohort. In addition, changes in global network metrics from baseline to the 6-month follow-up were compared between responder and relapser using independent-samples *t-*tests or Mann-Whitney *U* tests.

## 3 Results

### 3.1 Demographic and clinical data

Twenty-nine patients with a diagnosis of TD-PD were treated with VIM-MRgFUS. However, 4 patients did not complete the follow-up, and an additional 4 patients were excluded because of poor image quality. Consequently, 21 patients were included in the final analysis (18 males and 3 females). Demographic and clinical characteristics did not differ significantly between the included and excluded patients (all *p* > 0.05; Table 1 of Supplementary Data 1). Detailed demographic information for the included subjects is presented in Table 1.

**Table 1:** Demographic data of TD-PD patients.

Characteristics	TD-PD Patients (*n* = 21)
Age	61.76 ± 11.85
Sex: Male/female	18/3
Handedness: Right/non-right-handed	19/2
Age of onset	55.45 ± 12.47
Disease duration	5.72 ± 2.61
Family history: Yes/no	5/16
Treatment location VIM: Left/right	13/8

**Fig. 1:**
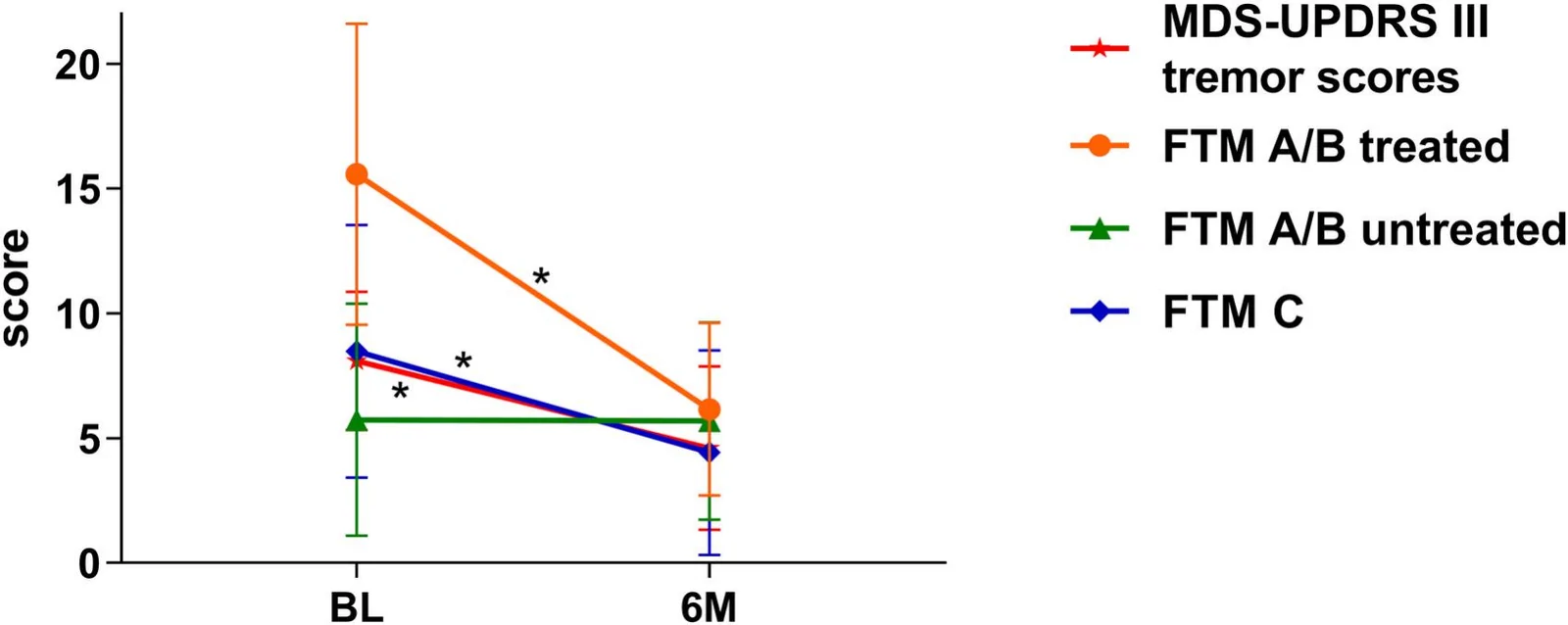
Changes in clinical scores from baseline (BL) to 6-month (6 M) follow-up after MRgFUS. Error bars represent standard deviations, and asterisks indicate significant differences (*p < 0.05).

Tremor scores on the treated side decreased significantly after MRgFUS. Specifically, MDS-UPDRS III tremor scores decreased from baseline (BL: M = 8.10, SD = 2.77) to 6 months (6 M, M = 4.60, SD = 3.27; *t* = 3.346, *p* = 0.009). The FTM A/B scores decreased from baseline (BL: M = 15.58, SD = 6.03) to 6 months (6 M, M = 6.16, SD = 3.45, *t* = 5.925, *p* < 0.001). In contrast, no significant change was observed on the non-treated side (BL: M = 5.74, SD = 4.64; 6 M: M = 5.68, SD = 3.96; *t* = 0.077, *p* = 0.940). The FTM C showed a significant decrease from BL (M = 8.47, SD = 5.06) to 6 M (M = 4.42, SD = 4.10; *t* = 4.731, *p* < 0.001; Fig. 1). In addition, only 19 patients with complete tremor scores at both baseline, 3-day and 6-month follow-up were included in the responder analysis. Two patients were excluded because tremor improvement could not be calculated due to missing MDS-UPDRS III tremor and FTM A/B tremor scores (Availability of clinical measures is provided in Table 2 of Supplementary Data 1). All 19 patients included in the responder analysis demonstrated ≥50% tremor improvement 3 days after MRgFUS. At 6-month follow-up, 10 patients maintained ≥50% tremor improvement and were classified as responder, and 9 patients showed <50% tremor improvement and were classified as relapser. Among patients with available MDS-UPDRS III tremor scores at 6-month follow-up, tremor improvement in FTM A/B was strongly correlated with tremor improvement in MDS-UPDRS III (*r* = 0.890, *p* = 0.001).

**Table 2:** Inter-modular connectivity.

	BL (mean ± SD)	6 M (mean ± SD)	*p* (uncorr.)	*p* (FDR)
module1&2	625.57 ± 194.74	647.10 ± 145.30	0.609	0.790
module1&3	401.05 ± 119.03	360.71 ± 148.28	0.256	0.790
module1&4	85.81 ± 56.74	79.95 ± 53.45	0.685	0.790
module1&5	139.43 ± 38.12	146.90 ± 71.15	0.648	0.790
module1&6	31.00 ± 30.91	29.57 ± 35.70	0.494	0.790
module2&3	536.00 ± 213.31	488.81 ± 179.54	0.394	0.790
module2&4	83.43 ± 68.38	65.24 ± 46.97	0.328	0.790
module2&5	110.52 ± 40.77	143.76 ± 99.54	0.212	0.790
module2&6	46.76 ± 43.64	69.19 ± 47.38	0.074*	0.615
module3&4	162.81 ± 90.72	177.86 ± 152.04	0.603	0.790
module3&5	95.90 ± 44.84	97.95 ± 44.41	0.847	0.908
module3&6	15.38 ± 17.41	15.57 ± 14.37	0.962	0.962
module4&5	35.57 ± 21.66	50.67 ± 40.94	0.082*	0.615
module4&6	12.33 ± 18.53	11.76 ± 20.53	0.588	0.790
module5&6	10.86 ± 13.56	12.71 ± 12.76	0.457	0.790

Inter-modular functional connectivity before and after MRgFUS treatment is presented, with module pairs exhibiting trend-level increases at 6 months highlighted (*p < 0.1).

### 3.2 Alterations of global and nodal topological properties

**Fig. 2:**
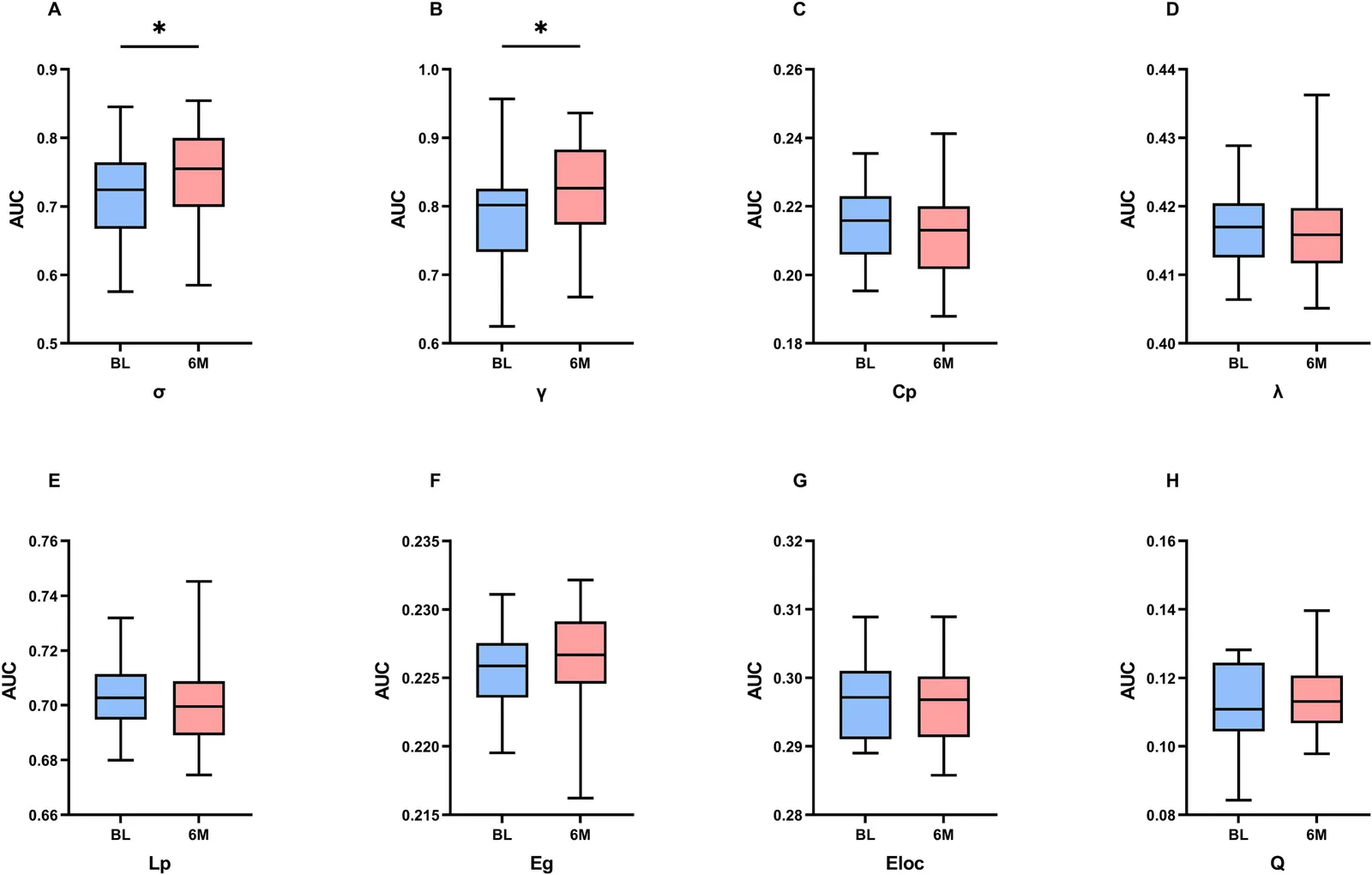
Changes in the AUC of global topological properties from BL to 6 M are shown. Asterisks indicate significant differences (*p < 0.05). σ and γ showed non-normal distribution and were compared using the Wilcoxon signed-rank test. Abbreviations: AUC, area under the curve; σ, small-worldness; γ, normalized clustering coefficient; Cp, clustering coefficient; λ, normalized characteristic path length; Lp, characteristic path length; Eg, global efficiency; Eloc, local efficiency; Q, modularity.

At a sparsity threshold of 0.05, the averaged degree of the network was 12.61, exceeding the required minimum value of 2 × log(273) = 11.22. Small-world properties (σ > 1.1) were consistently observed in both baseline and 6-month networks across sparsity thresholds from 0.05 to 0.44 (step = 0.01). Accordingly, network metrics were quantified across this threshold range. The AUC of σ (BL: 0.72, IQR 0.67–0.76 vs. 6 M: 0.75, IQR 0.70–0.80, *p* < 0.05) and γ (BL: 0.80, IQR 0.73–0.83 vs. 6 M: 0.83, IQR 0.77–0.88, *p* < 0.05) significantly increased after MRgFUS compared with baseline (Fig. 2A&B). In contrast, no significant differences were observed in the AUC of λ, Lp, Cp, Eloc, Eg, or Q (all *p* > 0.05; Fig. 2C-H). The temporal evolution of global network metrics, including the postoperative 3-day assessment, showed that several metrics experienced an initial postoperative decrease followed by recovery at the 6-month follow-up (Fig. 1 of Supplementary Data 1). Nodal topological metrics, including betweenness centrality, degree centrality, and nodal efficiency, did not show significant differences in any brain regions (all *p* > 0.05, FDR-corrected; Table 3 of Supplementary Data 1).

**Table 3:** Intra-modular connectivity.

	BL (mean ± SD)	6 M (mean ± SD)	*p* (uncorr.)	*p* (FDR)
module1	733.71 ± 173.51	677.67 ± 127.12	0.118	0.236
module2	1286.14 ± 222.20	1340.14 ± 244.08	0.373	0.373
module3	707.33 ± 136.26	665.10 ± 138.35	0.291	0.373
module4	137.14 ± 37.43	156.00 ± 53.90	0.106	0.236
module5	99.76 ± 24.97	116.00 ± 33.85	0.052*	0.236
module6	77.48 ± 20.62	81.33 ± 16.93	0.344	0.373

Intra-modular functional connectivity before and after MRgFUS treatment is presented. Module 5 showing a trend-level increase at 6 months is highlighted (*p < 0.1).

### 3.3 Robustness analysis

LOSO analyses demonstrated that the increase in γ remained significant after sequential exclusion of each participant, while the increase in σ remained significant in nearly all iterations, with only two exclusions resulting in borderline significance (maximum *p* = 0.050; Table 4 of Supplementary Data 1). No significant correlations were observed between the changes in σ and γ and individual mean FD (all *p* > 0.05; Table 5 of Supplementary Data 1).

### 3.4 Modular organization of functional networks

**Fig. 3:**
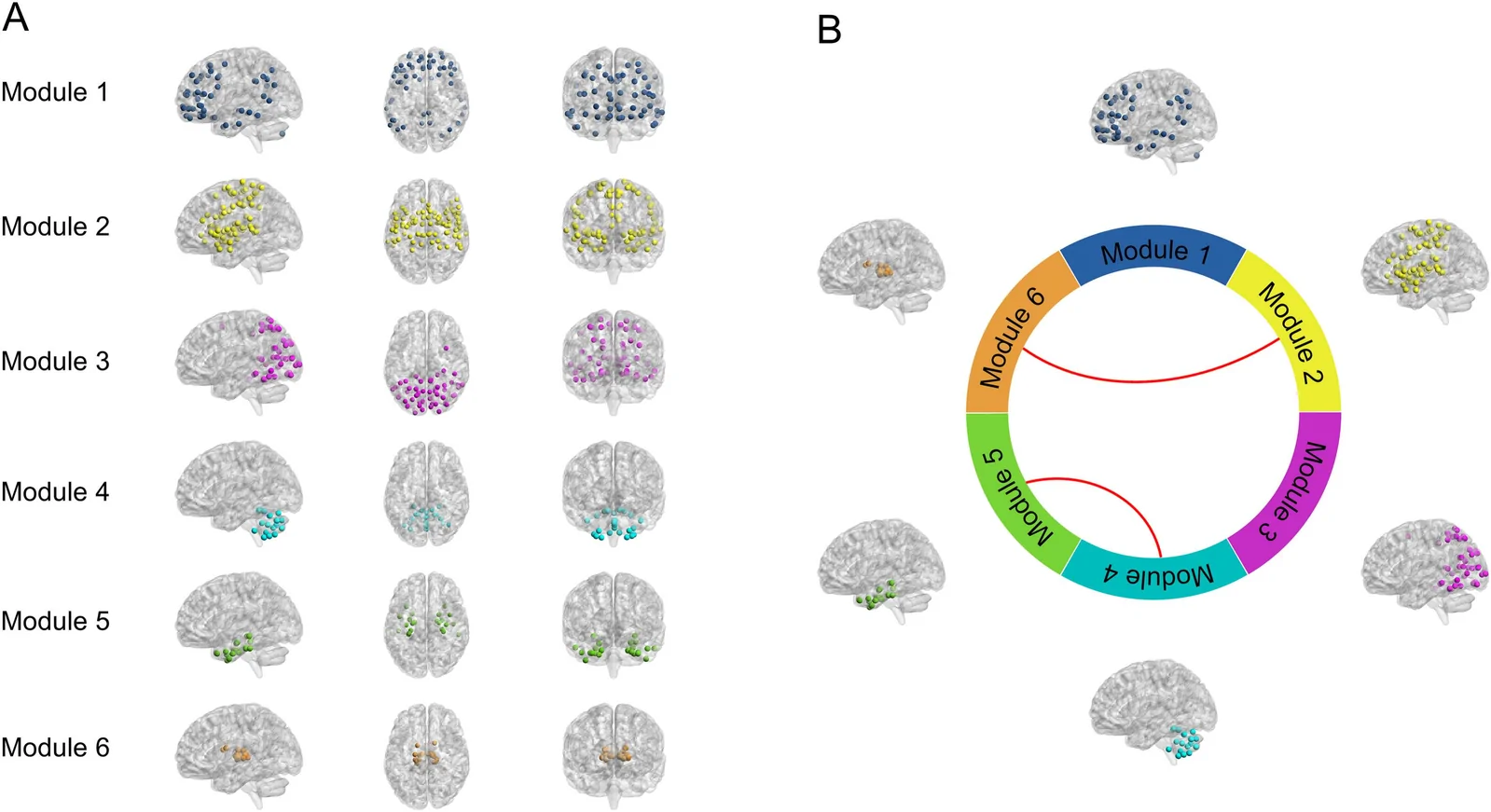
Modular organization of functional networks. (A) The spatial distribution of the six modules is visualized across sagittal, axial, and coronal planes. (B) The six modules are visualized using a circular layout, where red connections reflect a tendency toward increased interactions between modules.

Modular analysis identified six functional modules (Q = 0.434), including Module 1 (fronto-temporo-parietal, 61 ROIs), Module 2 (cortico-basal ganglia, 92 ROIs), Module 3 (occipito-temporo-parietal, 53 ROIs), Module 4 (cerebellar, 26 ROIs), Module 5 (temporal-hippocampal, 23 ROIs), and Module 6 (thalamic, 18 ROIs) (Fig. 3A). The overall modular organization was largely preserved across sparsity thresholds of 0.13 and 0.17, with only modest changes in module size (Fig. 2 of Supplementary Data 1).

No significant interactions between or within modules were observed at 6 months relative to baseline after FDR correction (*p* > 0.05, FDR-corrected; Table 2, Table 3), although trend-level increases were observed in the interaction between module 2 and module 6 (*p* = 0.074, uncorrected), and between module 4 and module 5 (*p* = 0.082, uncorrected; Fig. 3B). Additionally, the interaction within module 5 showed a trend-level increase (*p* = 0.052, uncorrected; Table 3). There were no significant changes in participation coefficient, betweenness centrality, degree centrality, and nodal efficiency for these four modules in any brain region (all *p* > 0.05, FDR-corrected; Table 6 of Supplementary Data 1).

### 3.5 Structural modular connectivity

In the structural network, no significant changes in inter-modular or intra-modular connectivity strength were observed at 6 months compared with baseline after FDR correction (all *p* > 0.05; Tables 7 and 8 of Supplementary Data 1). Trend-level decreases were observed in the connectivity strength between module 1 and module 4 (*p* = 0.092, uncorrected), module 2 and module 4 (*p* = 0.033, uncorrected), and module 2 and module 6 (*p* = 0.020, uncorrected).

### 3.6 Functional reorganization in responder

**Fig. 4:**
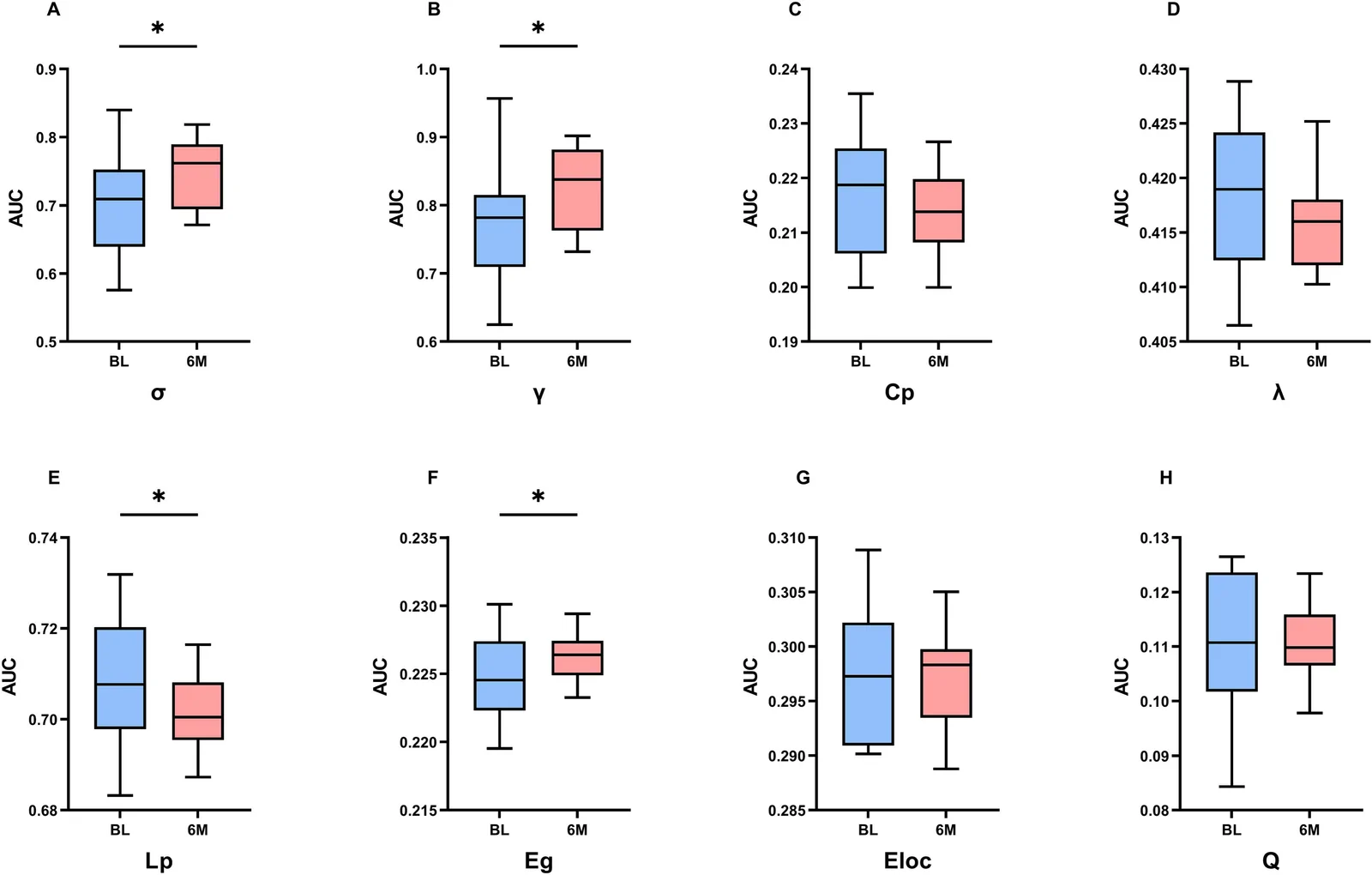
Changes in the AUC of global topological properties from BL to 6 M in responder are shown. Asterisks indicate significant differences (*p < 0.05). σ, γ, Lp, and Eg showed non-normal distribution and were compared using the Wilcoxon signed-rank test.

In responder, the AUC of σ (BL: 0.71, IQR 0.64–0.75 vs. 6 M: 0.76, IQR 0.69–0.79, *p* < 0.05; Fig. 4A) and γ (BL: 0.78, IQR 0.71–0.82 vs. 6 M: 0.84, IQR 0.76–0.88, *p* < 0.05; Fig. 4B) and Eg (BL: 0.2245, IQR 0.2223–0.2274 vs. 6 M: 0.2264, IQR 0.2249–0.2274, *p* < 0.05; Fig. 4F) increased significantly, whereas Lp (BL: 0.7077, IQR 0.6978–0.7203 vs. 6 M: 0.7005, IQR 0.6954–0.7081, *p* < 0.05; Fig. 4E) decreased significantly at 6 months post-MRgFUS compared to baseline. No significant changes were observed in the AUC of λ, Cp, Eloc, or Q (all *p* > 0.05; Fig. 4C, D, G, H).

Nodal measures showed no significant changes (all *p* > 0.05, FDR-corrected). Modular interactions showed a trend-level increase between module 4 and module 5 (*p* = 0.037, uncorrected). Nodal metrics within these modules, including participation coefficient, betweenness centrality, degree centrality, and nodal efficiency, remained unchanged (all *p* > 0.05, FDR-corrected; detailed results can be found in Supplementary Data 2).

### 3.7 Functional reorganization in relapser

In relapser, neither global nor nodal topological properties exhibited significant changes at 6 months relative to baseline. Modular interactions were also non-significant, although trend-level increases were noted between module 2 and module 6 (*p* = 0.082, uncorrected), trend-level increases within module 4 (*p* = 0.030, uncorrected), and trend-level decreases within module 1 (*p* = 0.018, uncorrected). No nodes within these modules showed significant changes in participation coefficient, betweenness centrality, degree centrality, and nodal efficiency. Additionally, the changes in all global network metrics did not differ significantly between responder and relapser (all *p* > 0.05; detailed results can be found in Supplementary Data 3).

### 3.8 Association between functional reorganization properties and clinical improvement

**Fig. 5:**
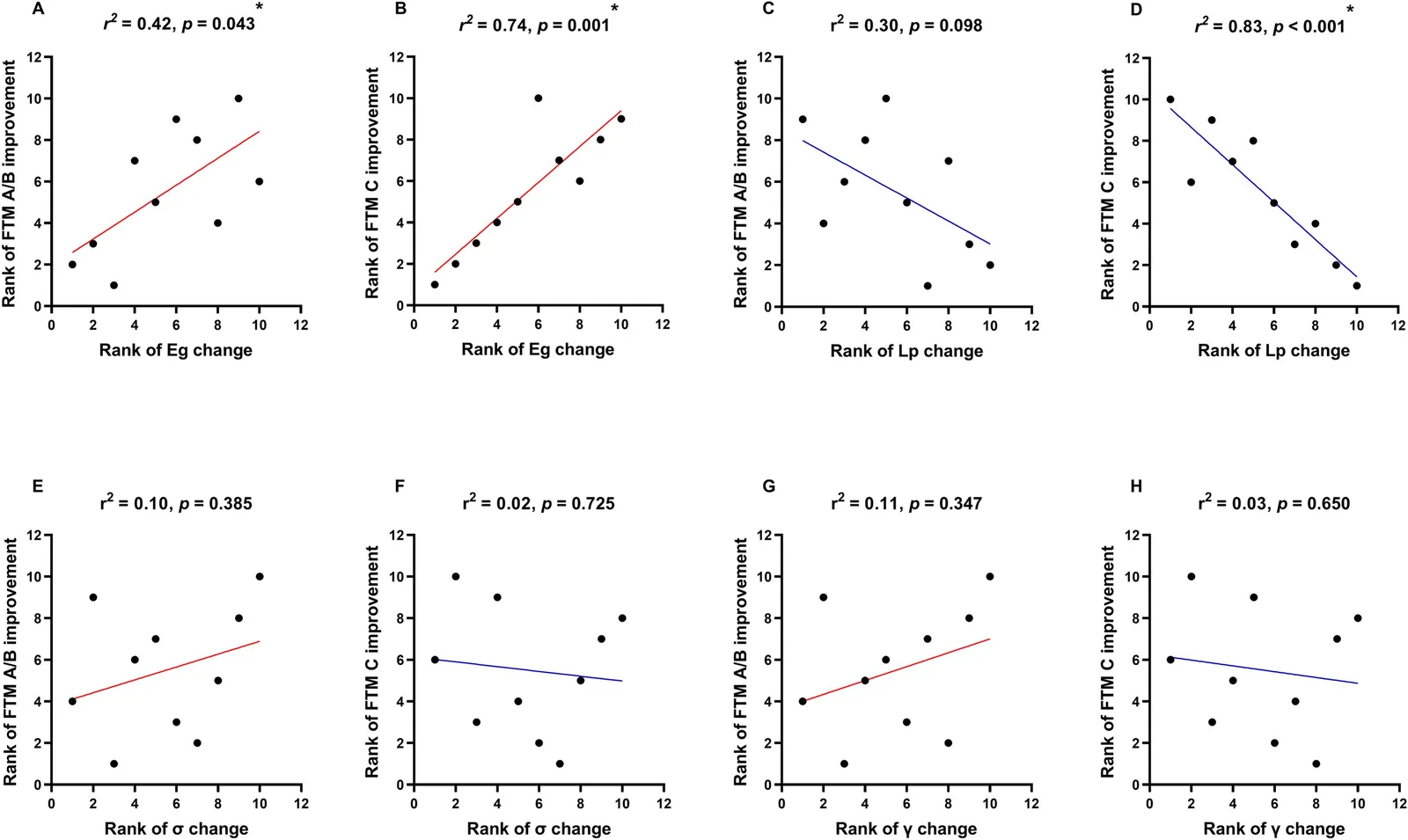
Correlations between global topological property changes and FTM improvement in responder. Data are displayed as ranks to reflect Spearman's rank correlation. Asterisks indicate significant differences (*p < 0.05).

Significant associations were found in responder only. Specifically, changes in Eg were positively correlated with improvement in FTM A/B (r^2^ = 0.42, *p* < 0.05, Fig. 5A) and FTM C (r^2^ = 0.74, *p* = 0.001, Fig. 5B), and changes in Lp were negatively correlated with improvement in FTM C (r^2^ = 0.83, *p* < 0.001, Fig. 5D). However, no significant correlations were found between changes in σ and γ and clinical improvement (all *p* < 0.05, Fig. 5E-H). Additionally, no significant correlations were found between changes in σ and γ and clinical improvement across overall cohort (all *p* > 0.05).

## 4 Discussion

In this study, we investigated the effects of MRgFUS thalamotomy on functional brain networks in TD-PD. Across all patients, MRgFUS led to increases in small-worldness and normalized clustering coefficient, along with trend-level reconfiguration of interactions between the cortical-basal ganglia and thalamic modules, as well as between the cerebellar and temporal-hippocampal modules. In responder, more pronounced changes were observed in global network integration, reflected by increased global efficiency and reduced characteristic path length, which were significantly associated with clinical improvement.

In the human brain, small-world organization supposedly supports efficient information processing while minimizing wiring cost, reflecting an economical network architecture that facilitates both specialized local processing and long-range communication (Bassett and Bullmore, 2017). Disruptions of small-world topology have been reported in PD (Suo et al., 2022), characterized by reduced clustering coefficient and network efficiency, along with increased characteristic path length, suggesting a shift away from an optimal small-world organization. In whole-cohort analyses, σ and γ significantly improved, while Lp, Cp, Eg, Eloc, and λ showed no significant changes, suggesting that MRgFUS may preferentially enhance relative clustering without substantially altering large-scale global integration. This pattern indicates a partial reorganization toward a more small-world-like architecture, rather than a complete normalization of global network topology. A comparable pattern of network reorganization has been observed in ET (Liu et al., 2026). Although ET and PD differ in their underlying pathophysiology, they share overlapping CTC circuits that are critically involved in tremor generation (Raethjen and Deuschl, 2012; Dirkx and Bologna, 2022). Therefore, the changes in σ and γ observed across the two disorders may reflect common network-level responses to thalamotomy. Nevertheless, because neither metric was associated with clinical improvement in either cohort, it remains possible that the observed changes reflect a general network response to focal VIM lesioning.

In addition, a longitudinal structural network study by Lin et al. (Lin et al., 2021) reported a U-shaped trajectory of multiple topological metrics following MRgFUS. In the present study, the temporal evolution of global network metrics showed that several metrics exhibited a similar U-shaped trajectory, with an initial postoperative decrease followed by recovery at the 6-month follow-up, suggesting that MRgFUS-induced network reorganization evolves over time. However, these early postoperative findings should be interpreted with caution, as postoperative edema can transiently enlarge the lesion, potentially altering functional connectivity and influencing network metrics during the acute stage (Jang et al., 2016; Keil et al., 2020).

Modularity was used to quantify the degree of functional segregation of the brain network (Rubinov and Sporns, 2010). Previous studies investigating modularity in PD have reported mixed findings. While some studies reported no significant difference in modularity between PD patients and healthy controls (Ma et al., 2017; Suo et al., 2022), one study reported increased modularity in PD (Baggio et al., 2014). In our study, modularity did not show significant changes six months after MRgFUS, indicating that the overall segregation of functional modules remained stable following the intervention. Within this preserved modular framework, six functional modules were identified, including fronto-temporo-parietal, cortical-basal ganglia, occipito-temporo-parietal, cerebellar, temporal-hippocampal, and thalamic modules. This modular architecture provided a basis for investigating modular interactions after MRgFUS.

We observed trend-level increases in connectivity between the cortical-basal ganglia module and the thalamic module. The cortical-basal ganglia module largely overlaps with regions comprising the basal ganglia-cortical circuit, and the thalamic module represents a key component of the CTC circuit (Dirkx and Bologna, 2022). According to the dimmer-switch hypothesis (Helmich et al., 2012), the maladaptive interactions between these two circuits contribute to the initiation and propagation of PD tremor. Abnormal connectivity between these two circuits in PD has already been reported. Agosta et al. (Agosta et al., 2014) found decreased functional connectivity within striatal and thalamic regions in PD patients compared with early PD and healthy controls, indicating disrupted basal ganglia-thalamic communication. Importantly, accumulating evidence suggests that MRgFUS can modulate key components of tremor-related circuits beyond the local lesion site. Stanziano et al. (Stanziano et al., 2021) observed increased functional connectivity between the pallidum and the dentate nucleus following MRgFUS, and Tani et al. (Tani et al., 2022) reported increased connectivity between the thalamus and the dorsal premotor cortex after MRgFUS. In contrast, the structural modular analysis demonstrated an opposite trend. Specifically, we observed trend-level decreases in structural connectivity strength between the cortical-basal ganglia and thalamic modules, as well as between the cortical-basal ganglia and cerebellar modules. These findings may reflect persistent disruption of structural pathways within the tremor network following lesioning. The increased functional connectivity between the cortical-basal ganglia and thalamic modules may reflect compensatory network reorganization in response to persistent structural disconnection.

Additionally, a trend toward increased interaction between the cerebellar module and the temporal-hippocampal module was observed. The cerebellum is a key component of tremor-related networks and plays a critical role in the generation and modulation of tremor in PD (Zhong et al., 2022). Increasing evidence suggests that cerebellar functions extend beyond motor control, contributing indispensably to non-motor domains such as cognition and emotion. Anatomical studies support this view, indicating that the cerebellum possesses widespread connections with cortical regions involved in higher-order brain functions (Mastrangelo et al., 2024). The temporal lobe and hippocampal system are critically involved in memory, learning, and emotion (Zhu et al., 2019). Recent work has demonstrated bidirectional functional connectivity between the cerebellum and hippocampus, which is critical for spatial navigation and temporally dependent tasks (Yu and Krook-Magnuson, 2015). In PD, aberrant cerebellar-temporal-hippocampal pathways have been associated with non-motor symptoms. For instance, reduced functional connectivity between the non-motor cerebellum and hippocampus has been reported in early, drug-naïve PD patients (Jiang et al., 2023), and the strength of cerebellar-medial temporal connections has been positively correlated with depression and anxiety scores (He et al., 2025). Further research is needed to substantiate the functional impact of these findings, given that non-motor symptoms were not extensively acquired in this cohort.

Although whole-cohort analyses revealed changes in a subset of global metrics, such aggregate results may mask inter-individual variability in treatment response. Previous studies have highlighted the heterogeneity in clinical outcomes following MRgFUS in PD (Tian et al., 2023; Peters et al., 2024; Braccia et al., 2025). Notably, a recent study reported that younger age and smaller lesion volume were associated with a higher risk of relapse after MRgFUS, underscoring the importance of individual biological and procedural factors in determining treatment efficacy (Braccia et al., 2025). In our study, significant alterations in global topological properties were observed only in responder, whereas relapser exhibited no significant changes in global or nodal network measures. In responder, changes in global efficiency and characteristic path length were significantly correlated with clinical improvement, suggesting that effective symptom relief following MRgFUS is closely linked to enhanced global integration within functional brain networks. These results suggest that MRgFUS may induce a large-scale reconfiguration of functional brain networks, facilitating more efficient information transfer across distributed regions and thereby supporting clinical improvement. However, the absence of significant findings in relapser should be interpreted cautiously, as limited statistical power may have obscured treatment-related changes in network organization. The lack of significant differences between responder and relapser should not be interpreted as evidence of similar network reorganization patterns, given the limited power of the subgroup analyses.

Several limitations should be acknowledged in the present study. The sample size was relatively small, and dividing patients into responder and relapser groups further reduced the number of subjects per group, which may have limited statistical power, particularly for subgroup analyses. Additionally, although fMRI data were acquired at baseline, 3-day and 6-month follow-up, the lack of intermediate (e.g., 1- or 3-month) and longer-term (e.g., ≥12-month) follow-up prevented continuous assessment of the dynamic evolution and long-term stability of clinical benefit and functional network changes, limiting the interpretation of the observed imaging-clinical correlations. Furthermore, the study was conducted in a single cohort without a control or sham group, limiting causal inference about observed functional network changes. Moreover, the use of binarized networks may lead to the loss of edge weight information, while modular analysis at a single sparsity threshold may be sensitive to threshold selection. In addition, correlation analyses were based on FTM improvement because follow-up MDS-UPDRS data were unavailable in several patients, which may have limited comparability across clinical outcome measures. Finally, although one-tailed tests were applied to the global network metrics based on a priori hypotheses, this approach may increase the risk of Type I error (Chen et al., 2019; Lindquist and Mejia, 2015), warranting cautious interpretation of the corresponding global findings.

In conclusion, our study indicates that MRgFUS thalamotomy in TD-PD may promote partial reorganization of small-world properties and enhance global functional integration in responder. At the cohort level, MRgFUS increased small-worldness and normalized clustering coefficient, together with trend-level reconfiguration of interactions between the cortical-basal ganglia and thalamic modules, as well as between the cerebellar and temporal-hippocampal modules. Importantly, more pronounced global network reorganization emerged specifically in responder, characterized by increased global efficiency and reduced characteristic path length, both of which were significantly associated with clinical improvement. These findings provide novel insights into the network-level changes induced by MRgFUS in TD-PD. Future research with larger, well-controlled longitudinal cohorts is required to confirm the robustness of these findings.

## CRediT authorship contribution statement

**Jinlong Liu:** Writing – original draft, Methodology, Investigation, Conceptualization. **Veronika Purrer:** Writing – review & editing, Data curation. **Jonas Krauss:** Writing – review & editing, Data curation. **Valeri Borger:** Writing – review & editing, Data curation. **Markus Essler:** Writing – review & editing. **Alexander Radbruch:** Writing – review & editing. **Ullrich Wüllner:** Writing – review & editing, Funding acquisition. **Neeraj Upadhyay:** Writing – review & editing, Supervision, Methodology. **Henning Boecker:** Writing – review & editing, Supervision, Funding acquisition.

## Ethics declaration

### Informed consent and patient details

Written informed consent to take part in the study and to publish the article has been obtained from all participants or their legal representatives. The privacy rights of participants have been observed.

### Studies in human

This study was performed in compliance with relevant laws, regulatory frameworks and guidelines where the research took place.

This study was approved by the Ethics Committee of the University Hospital Bonn.

(Approval No. 314/18)

## Funding

The MRgFUS facility was funded by the German Research Foundation (INST 1172/64–1) and the Medical Faculty of the University of Bonn. Open access funding was enabled and organized by Project DEAL.

## Declaration of competing interest

The authors declare that they have no known competing financial interests or personal relationships that could have appeared to influence the work reported in this paper.

## Data Availability

The data that support the findings of this study are available from the corresponding author upon reasonable request.
