# Supplementary material for: Functional network reorganization after magnetic resonance-guided focused ultrasound thalamotomy in tremor-dominant Parkinson's disease patients
Source: Neuroimage Clin. 2026 Sep 5;52:104058. doi: 10.1016/j.nicl.2026.104058 (PMC13573704; doi:10.1016/j.nicl.2026.104058)
Supplement: Supplementary file 1 [file mmc1.docx]

**Supplementary Methods**

**Diffusion MRI acquisition**

Diffusion-weighted MRI was acquired using a single-shot spin-echo echo planar imaging (EPI) sequence with the following parameters: echo time (TE) = 67 ms, repetition time (TR) = 7780, flip angle = 90°, voxel size = 2 × 2 × 2 mm³, 61 diffusion-weighted directions (b = 1200 s/mm²), and one non-diffusion-weighted (b0) image. An additional b0 image with reversed phase-encoding polarity was acquired for susceptibility-induced distortion correction.

**Preprocessing and network construction**

Diffusion MRI data were preprocessed using Mrtrix3 (<https://www.mrtrix.org/>). Image denoising was performed using the Marchenko-Pastur principal component analysis (MP-PCA) method, followed by Gibbs ringing correction. Motion and susceptibility-induced distortion corrections were performed in a single step using FSL's TOPUP (Andersson et al. 2003) and eddy (Andersson and Sotiropoulos 2016).

Anatomically constrained probabilistic tractography was performed following T1-weighted image parcellation using FreeSurfer 7.4.1(<https://surfer.nmr.mgh.harvard.edu/>) and registration to diffusion space. The structural connectivity matrices were constructed using 273 regions of interest (ROIs) defined by the Human Brainnetome Atlas. Connection strengths were normalized by the inverse node volume.

**Structural modular connectivity analysis**

To complement the functional modular analysis, the same modular partition identified from the functional network was applied to the structural connectivity matrices. Average inter-modular and intra-modular connectivity strengths were calculated. Statistical analyses were performed using the same procedures as those described for the functional modular analysis, including correction for multiple comparisons using the false discovery rate (FDR).

**Reference**

Andersson JL et al., 2003. How to correct susceptibility distortions in spin-echo echo-planar images: application to diffusion tensor imaging. NeuroImage 20, 870-888. <https://doi.org/10.1016/s1053-8119(03)00336-7>.

Andersson JLR, Sotiropoulos SN, 2016. An integrated approach to correction for off-resonance effects and subject movement in diffusion MR imaging. NeuroImage 125, 1063-1078. <https://doi.org/10.1016/j.neuroimage.2015.10.019>.
