# Supplementary material for: Functional network reorganization after magnetic resonance-guided focused ultrasound thalamotomy in tremor-dominant Parkinson's disease patients
Source: Neuroimage Clin. 2026 Sep 5;52:104058. doi: 10.1016/j.nicl.2026.104058 (PMC13573704; doi:10.1016/j.nicl.2026.104058)
Supplement: Supplementary file 2 [file mmc2.docx]

**Fig. 1.** Temporal evolution of global network metrics following MRgFUS. Data are presented as mean ± SD of the AUC for global metrics at baseline (BL), 3-day (3D), and 6-month follow-up (6M).

**Fig. 2.** Modular organization of functional brain networks at different sparsity thresholds. The spatial distribution of the six functional modules is shown in sagittal, axial, and coronal views. (A) Modular organization at a sparsity threshold of 0.13, including Module 1 (53 ROIs), Module 2 (102 ROIs), Module 3 (45 ROIs), Module 4 (30 ROIs), Module 5 (27 ROIs), and Module 6 (16 ROIs). (B) Modular organization at a sparsity threshold of 0.17, including Module 1 (53 ROIs), Module 2 (96 ROIs), Module 3 (50 ROIs), Module 4 (28 ROIs), Module 5 (27 ROIs), and Module 6 (19 ROIs).

**Table 1**

Comparison of demographic and clinical characteristics between included and excluded patients

| Variable | Included (n=21) | Excluded (n=8) | *p* value |
| --- | --- | --- | --- |
| Age (years) | 61.76±11.85 | 62.00±10.12 | 0.899 |
| Female sex, n (%) | 3 (14.29%) | 1 (12.50%) | 1.000 |
| Age of onset (years) | 55.45±12.47 | 57.67±10.78 | 0.548 |
| Disease duration (years) | 5.72±2.61 | 4.33±2.73 | 0.282 |
| FTM A/B at baseline | 15.58±6.03 | 11.80±2.39 | 0.175 |

**Table 2**

Availability of clinical measures for tremor on the treated side

| Patient ID | MDS-UPDRS III baseline | MDS-UPDRS III 3 days | MDS-UPDRS III 6 months | FTM baseline | FTM 3 days | FTM 6 months |
| --- | --- | --- | --- | --- | --- | --- |
| 1 | ✓ | ✓ | ✓ | ✓ | ✓ | ✓ |
| 2 | ✓ | ✓ | ✓ | ✓ | ✓ | ✓ |
| 3 | ✓ | ✓ | ✓ | ✓ | ✓ | ✓ |
| 4 | ✓ | ✓ | ✓ | ✓ | ✓ | ✓ |
| 5 | ✓ | ✓ | ✓ | ✓ | ✓ | ✓ |
| 6 | ✓ | ✓ | ✓ | ✓ | ✓ | ✓ |
| 7 | ✓ | ✓ | ✓ | ✓ | ✓ | ✓ |
| 8 | ✓ | ✓ | ✓ | ✓ | ✓ | ✓ |
| 9 | ✓ | ✓ | ✓ | ✓ | ✓ | ✓ |
| 10 | ✓ | ✓ | ✓ | ✓ | ✓ | ✓ |
| 11 | – | – | – | ✓ | ✓ | ✓ |
| 12 | – | – | – | ✓ | ✓ | ✓ |
| 13 | – | – | – | ✓ | ✓ | ✓ |
| 14 | – | – | – | ✓ | ✓ | ✓ |
| 15 | – | – | – | ✓ | ✓ | ✓ |
| 16 | – | – | – | ✓ | ✓ | ✓ |
| 17 | – | – | – | ✓ | ✓ | ✓ |
| 18 | – | – | – | ✓ | ✓ | ✓ |
| 19 | – | – | – | ✓ | ✓ | ✓ |
| 20 | – | – | – | – | – | – |
| 21 | – | – | – | – | – | – |

✓, available; –, unavailable.

**Table 3**

Changes in the AUC of nodal topological properties between BL and 6M

| Region | Node ID ^a^ | *p* value ^b^ | | |
| --- | --- | --- | --- | --- |
|  |  | Betweenness centrality | Degree centrality | Nodal efficiency |
| Superior frontal gyrus(SFG) | SFG_L_7_1 | 0.934 | 0.982 | 0.987 |
|  | SFG_R_7_1 | 0.927 | 0.662 | 0.655 |
|  | SFG_L_7_2 | 0.927 | 0.858 | 0.956 |
|  | SFG_R_7_2 | 0.934 | 0.751 | 0.833 |
|  | SFG_L_7_3 | 0.927 | 0.894 | 0.987 |
|  | SFG_R_7_3 | 0.927 | 0.894 | 0.987 |
|  | SFG_L_7_4 | 0.914 | 0.772 | 0.675 |
|  | SFG_R_7_4 | 0.914 | 0.894 | 0.854 |
|  | SFG_L_7_5 | 0.914 | 0.904 | 0.828 |
|  | SFG_R_7_5 | 0.914 | 0.891 | 0.810 |
|  | SFG_L_7_6 | 0.927 | 0.784 | 0.810 |
|  | SFG_R_7_6 | 0.945 | 0.718 | 0.675 |
|  | SFG_L_7_7 | 0.989 | 0.992 | 0.988 |
|  | SFG_R_7_7 | 0.927 | 0.982 | 0.988 |
| Middle frontal gyrus(MFG) | MFG_L_7_1 | 0.934 | 0.894 | 0.885 |
|  | MFG_R_7_1 | 0.927 | 0.864 | 0.882 |
|  | MFG_L_7_2 | 0.927 | 0.894 | 0.987 |
|  | MFG_R_7_2 | 0.927 | 0.982 | 0.988 |
|  | MFG_L_7_3 | 0.914 | 0.891 | 0.955 |
|  | MFG_R_7_3 | 0.914 | 0.812 | 0.854 |
|  | MFG_L_7_4 | 0.927 | 0.982 | 0.988 |
|  | MFG_R_7_4 | 0.927 | 0.883 | 0.854 |
|  | MFG_L_7_5 | 0.927 | 0.908 | 0.975 |
|  | MFG_R_7_5 | 0.870 | 0.891 | 0.885 |
|  | MFG_L_7_6 | 0.956 | 0.982 | 0.955 |
|  | MFG_R_7_6 | 0.927 | 0.894 | 0.933 |
|  | MFG_L_7_7 | 0.914 | 0.894 | 0.987 |
|  | MFG_R_7_7 | 0.934 | 0.891 | 0.955 |
| Inferior frontal gyrus(IFG) | IFG_L_6_1 | 0.948 | 0.812 | 0.854 |
|  | IFG_R_6_1 | 0.945 | 0.812 | 0.810 |
|  | IFG_L_6_2 | 0.914 | 0.706 | 0.655 |
|  | IFG_R_6_2 | 0.927 | 0.945 | 0.987 |
|  | IFG_L_6_3 | 0.914 | 0.894 | 0.882 |
|  | IFG_R_6_3 | 0.927 | 0.894 | 0.928 |
|  | IFG_L_6_4 | 0.927 | 0.891 | 0.885 |
|  | IFG_R_6_4 | 0.934 | 0.894 | 0.920 |
|  | IFG_L_6_5 | 0.934 | 0.418 | 0.358 |
|  | IFG_R_6_5 | 0.989 | 0.751 | 0.709 |
|  | IFG_L_6_6 | 0.934 | 0.440 | 0.358 |
|  | IFG_R_6_6 | 0.977 | 0.894 | 0.885 |
| Orbital gyrus(OrG) | OrG_L_6_1 | 0.927 | 0.982 | 0.988 |
|  | OrG_R_6_1 | 0.914 | 0.706 | 0.655 |
|  | OrG_L_6_2 | 0.934 | 0.982 | 0.988 |
|  | OrG_R_6_2 | 0.934 | 0.982 | 0.987 |
|  | OrG_L_6_3 | 0.978 | 0.371 | 0.358 |
|  | OrG_R_6_3 | 0.914 | 0.784 | 0.828 |
|  | OrG_L_6_4 | 0.953 | 0.904 | 0.988 |
|  | OrG_R_6_4 | 0.927 | 0.706 | 0.709 |
|  | OrG_L_6_5 | 0.934 | 0.812 | 0.714 |
|  | OrG_R_6_5 | 0.927 | 0.904 | 0.988 |
|  | OrG_L_6_6 | 0.914 | 0.845 | 0.854 |
|  | OrG_R_6_6 | 0.938 | 0.812 | 0.854 |
| Precentral gyrus(PrG) | PrG_L_6_1 | 0.914 | 0.662 | 0.655 |
|  | PrG_R_6_1 | 0.934 | 0.751 | 0.714 |
|  | PrG_L_6_2 | 0.945 | 0.746 | 0.655 |
|  | PrG_R_6_2 | 0.934 | 0.904 | 0.887 |
|  | PrG_L_6_3 | 0.927 | 0.649 | 0.631 |
|  | PrG_R_6_3 | 0.956 | 0.894 | 0.885 |
|  | PrG_L_6_4 | 0.914 | 0.992 | 0.955 |
|  | PrG_R_6_4 | 0.956 | 0.649 | 0.655 |
|  | PrG_L_6_5 | 0.914 | 0.349 | 0.293 |
|  | PrG_R_6_5 | 0.934 | 0.785 | 0.665 |
|  | PrG_L_6_6 | 0.914 | 0.649 | 0.608 |
|  | PrG_R_6_6 | 0.927 | 0.811 | 0.854 |
| Paracentral lobule(PCL) | PCL_L_2_1 | 0.996 | 0.649 | 0.655 |
|  | PCL_R_2_1 | 0.927 | 0.643 | 0.655 |
|  | PCL_L_2_2 | 0.927 | 0.718 | 0.698 |
|  | PCL_R_2_2 | 0.934 | 0.649 | 0.655 |
| Superior temporal gyrus(STG) | STG_L_6_1 | 0.927 | 0.649 | 0.655 |
|  | STG_R_6_1 | 0.914 | 0.839 | 0.817 |
|  | STG_L_6_2 | 0.934 | 0.894 | 0.882 |
|  | STG_R_6_2 | 0.927 | 0.894 | 0.833 |
|  | STG_L_6_3 | 0.996 | 0.718 | 0.655 |
|  | STG_R_6_3 | 0.927 | 0.982 | 0.987 |
|  | STG_L_6_4 | 0.914 | 0.349 | 0.358 |
|  | STG_R_6_4 | 0.927 | 0.894 | 0.955 |
|  | STG_L_6_5 | 0.914 | 0.711 | 0.655 |
|  | STG_R_6_5 | 0.914 | 0.904 | 0.956 |
|  | STG_L_6_6 | 0.977 | 0.746 | 0.817 |
|  | STG_R_6_6 | 0.927 | 0.982 | 0.955 |
| Middle temporal gyrus(MTG) | MTG_L_4_1 | 0.927 | 0.594 | 0.584 |
|  | MTG_R_4_1 | 0.989 | 0.718 | 0.782 |
|  | MTG_L_4_2 | 0.927 | 0.711 | 0.655 |
|  | MTG_R_4_2 | 0.870 | 0.649 | 0.608 |
|  | MTG_L_4_3 | 0.927 | 0.341 | 0.293 |
|  | MTG_R_4_3 | 0.914 | 0.349 | 0.358 |
|  | MTG_L_4_4 | 0.927 | 0.598 | 0.631 |
|  | MTG_R_4_4 | 0.816 | 0.473 | 0.399 |
| Inferior temporal gyrus(ITG) | ITG_L_7_1 | 0.914 | 0.649 | 0.584 |
|  | ITG_R_7_1 | 0.927 | 0.894 | 0.994 |
|  | ITG_L_7_2 | 0.927 | 0.894 | 0.885 |
|  | ITG_R_7_2 | 0.914 | 0.649 | 0.643 |
|  | ITG_L_7_3 | 0.996 | 0.982 | 0.975 |
|  | ITG_R_7_3 | 0.914 | 0.746 | 0.714 |
|  | ITG_L_7_4 | 0.914 | 0.746 | 0.655 |
|  | ITG_R_7_4 | 0.927 | 0.718 | 0.827 |
|  | ITG_L_7_5 | 0.927 | 0.649 | 0.655 |
|  | ITG_R_7_5 | 0.914 | 0.473 | 0.399 |
|  | ITG_L_7_6 | 0.958 | 0.894 | 0.885 |
|  | ITG_R_7_6 | 0.996 | 0.894 | 0.956 |
|  | ITG_L_7_7 | 0.996 | 0.731 | 0.682 |
|  | ITG_R_7_7 | 0.914 | 0.908 | 0.987 |
| Fusiform gyrus(FuG) | FuG_L_3_1 | 0.927 | 0.731 | 0.655 |
|  | FuG_R_3_1 | 0.927 | 0.982 | 0.988 |
|  | FuG_L_3_2 | 0.914 | 0.662 | 0.655 |
|  | FuG_R_3_2 | 0.927 | 0.726 | 0.655 |
|  | FuG_L_3_3 | 0.934 | 0.812 | 0.882 |
|  | FuG_R_3_3 | 0.914 | 0.890 | 0.885 |
| Parahippocampal gyrus(PhG) | PhG_L_6_1 | 0.731 | 0.486 | 0.399 |
|  | PhG_R_6_1 | 0.934 | 0.510 | 0.571 |
|  | PhG_L_6_2 | 0.936 | 0.894 | 0.833 |
|  | PhG_R_6_2 | 0.914 | 0.952 | 0.956 |
|  | PhG_L_6_3 | 0.620 | 0.982 | 0.988 |
|  | PhG_R_6_3 | 0.956 | 0.908 | 0.956 |
|  | PhG_L_6_4 | 0.927 | 0.649 | 0.644 |
|  | PhG_R_6_4 | 0.989 | 0.746 | 0.655 |
|  | PhG_L_6_5 | 0.914 | 0.349 | 0.358 |
|  | PhG_R_6_5 | 0.914 | 0.349 | 0.358 |
|  | PhG_L_6_6 | 0.914 | 0.706 | 0.655 |
|  | PhG_R_6_6 | 0.927 | 0.849 | 0.854 |
| Posterior superior temporal sulcus(pSTS) | pSTS_L_2_1 | 0.948 | 0.662 | 0.655 |
|  | pSTS_R_2_1 | 0.934 | 0.746 | 0.708 |
|  | pSTS_L_2_2 | 0.927 | 0.649 | 0.644 |
|  | pSTS_R_2_2 | 0.927 | 0.649 | 0.644 |
| Superior parietal lobule(SPL) | SPL_L_5_1 | 0.914 | 0.982 | 0.988 |
|  | SPL_R_5_1 | 0.870 | 0.718 | 0.698 |
|  | SPL_L_5_2 | 0.914 | 0.706 | 0.655 |
|  | SPL_R_5_2 | 0.927 | 0.718 | 0.655 |
|  | SPL_L_5_3 | 0.914 | 0.945 | 0.997 |
|  | SPL_R_5_3 | 0.914 | 0.649 | 0.655 |
|  | SPL_L_5_4 | 0.914 | 0.649 | 0.655 |
|  | SPL_R_5_4 | 0.914 | 0.731 | 0.714 |
|  | SPL_L_5_5 | 0.731 | 0.794 | 0.768 |
|  | SPL_R_5_5 | 0.927 | 0.706 | 0.655 |
| Inferior parietal lobule(IPL) | IPL_L_6_1 | 0.914 | 0.649 | 0.646 |
|  | IPL_R_6_1 | 0.914 | 0.473 | 0.399 |
|  | IPL_L_6_2 | 0.914 | 0.904 | 0.933 |
|  | IPL_R_6_2 | 0.914 | 0.751 | 0.794 |
|  | IPL_L_6_3 | 0.914 | 0.891 | 0.942 |
|  | IPL_R_6_3 | 0.927 | 0.418 | 0.358 |
|  | IPL_L_6_4 | 0.914 | 0.649 | 0.644 |
|  | IPL_R_6_4 | 0.927 | 0.711 | 0.655 |
|  | IPL_L_6_5 | 0.989 | 0.610 | 0.655 |
|  | IPL_R_6_5 | 0.914 | 0.349 | 0.358 |
|  | IPL_L_6_6 | 0.927 | 0.731 | 0.655 |
|  | IPL_R_6_6 | 0.927 | 0.894 | 0.966 |
| Precuneus(Pcun) | PCun_L_4_1 | 0.996 | 0.349 | 0.358 |
|  | PCun_R_4_1 | 0.914 | 0.349 | 0.358 |
|  | PCun_L_4_2 | 0.914 | 0.349 | 0.293 |
|  | PCun_R_4_2 | 0.914 | 0.418 | 0.358 |
|  | PCun_L_4_3 | 0.927 | 0.888 | 0.882 |
|  | PCun_R_4_3 | 0.914 | 0.649 | 0.655 |
|  | PCun_L_4_4 | 0.927 | 0.746 | 0.827 |
|  | PCun_R_4_4 | 0.927 | 0.649 | 0.655 |
| Postcentral gyrus(PoG) | PoG_L_4_1 | 0.914 | 0.649 | 0.559 |
|  | PoG_R_4_1 | 0.934 | 0.789 | 0.828 |
|  | PoG_L_4_2 | 0.914 | 0.784 | 0.687 |
|  | PoG_R_4_2 | 0.934 | 0.894 | 0.885 |
|  | PoG_L_4_3 | 0.870 | 0.784 | 0.655 |
|  | PoG_R_4_3 | 0.816 | 0.418 | 0.383 |
|  | PoG_L_4_4 | 0.928 | 0.812 | 0.892 |
|  | PoG_R_4_4 | 0.934 | 0.731 | 0.733 |
| Insular gyrus(INS) | INS_L_6_1 | 0.948 | 0.793 | 0.675 |
|  | INS_R_6_1 | 0.914 | 0.733 | 0.828 |
|  | INS_L_6_2 | 0.996 | 0.894 | 0.885 |
|  | INS_R_6_2 | 0.927 | 0.785 | 0.728 |
|  | INS_L_6_3 | 0.989 | 0.649 | 0.638 |
|  | INS_R_6_3 | 0.927 | 0.784 | 0.698 |
|  | INS_L_6_4 | 0.914 | 0.649 | 0.571 |
|  | INS_R_6_4 | 0.945 | 0.982 | 0.988 |
|  | INS_L_6_5 | 0.870 | 0.662 | 0.559 |
|  | INS_R_6_5 | 0.927 | 0.866 | 0.956 |
|  | INS_L_6_6 | 0.914 | 0.349 | 0.319 |
|  | INS_R_6_6 | 0.956 | 0.764 | 0.655 |
| Cingulate gyrus(CG) | CG_L_7_1 | 0.927 | 0.688 | 0.655 |
|  | CG_R_7_1 | 0.934 | 0.746 | 0.728 |
|  | CG_L_7_2 | 0.914 | 0.473 | 0.358 |
|  | CG_R_7_2 | 0.927 | 0.711 | 0.655 |
|  | CG_L_7_3 | 0.870 | 0.894 | 0.972 |
|  | CG_R_7_3 | 0.934 | 0.784 | 0.817 |
|  | CG_L_7_4 | 0.948 | 0.597 | 0.631 |
|  | CG_R_7_4 | 0.934 | 0.746 | 0.767 |
|  | CG_L_7_5 | 0.927 | 0.750 | 0.655 |
|  | CG_R_7_5 | 0.927 | 0.746 | 0.665 |
|  | CG_L_7_6 | 0.927 | 0.982 | 0.956 |
|  | CG_R_7_6 | 0.914 | 0.982 | 0.956 |
|  | CG_L_7_7 | 0.938 | 0.706 | 0.655 |
|  | CG_R_7_7 | 0.927 | 0.890 | 0.885 |
| MedioVentral occipital cortex(MVOcC) | MVOcC_L_5_1 | 0.934 | 0.982 | 0.988 |
|  | MVOcC_R_5_1 | 0.934 | 0.751 | 0.708 |
|  | MVOcC_L_5_2 | 0.914 | 0.784 | 0.854 |
|  | MVOcC_R_5_2 | 0.927 | 0.718 | 0.698 |
|  | MVOcC_L_5_3 | 0.914 | 0.962 | 0.956 |
|  | MVOcC_R_5_3 | 0.914 | 0.894 | 0.929 |
|  | MVOcC_L_5_4 | 0.927 | 0.812 | 0.854 |
|  | MVOcC_R_5_4 | 0.989 | 0.785 | 0.782 |
|  | MVOcC_L_5_5 | 0.927 | 0.772 | 0.827 |
|  | MVOcC_R_5_5 | 0.927 | 0.594 | 0.588 |
| Lateral occipital cortex(LOcC) | LOcC_L_4_1 | 0.927 | 0.737 | 0.683 |
|  | LOcC_R_4_1 | 0.927 | 0.751 | 0.698 |
|  | LOcC_L_4_2 | 0.958 | 0.649 | 0.655 |
|  | LOcC_R_4_2 | 0.914 | 0.688 | 0.655 |
|  | LOcC_L_4_3 | 0.934 | 0.718 | 0.655 |
|  | LOcC_R_4_3 | 0.948 | 0.785 | 0.714 |
|  | LOcC_L_4_4 | 0.927 | 0.883 | 0.885 |
|  | LOcC_R_4_4 | 0.914 | 0.982 | 0.987 |
|  | LOcC_L_2_1 | 0.914 | 0.649 | 0.655 |
|  | LOcC_R_2_1 | 0.914 | 0.894 | 0.895 |
|  | LOcC_L_2_2 | 0.914 | 0.688 | 0.655 |
|  | LOcC_R_2_2 | 0.934 | 0.546 | 0.465 |
| Amygdala(Amyg) | Amyg_L_2_1 | 0.914 | 0.418 | 0.358 |
|  | Amyg_R_2_1 | 0.914 | 0.751 | 0.655 |
|  | Amyg_L_2_2 | 0.870 | 0.349 | 0.319 |
|  | Amyg_R_2_2 | 0.914 | 0.649 | 0.631 |
| Hippocampus(Hipp) | Hipp_L_2_1 | 0.927 | 0.418 | 0.358 |
|  | Hipp_R_2_1 | 0.934 | 0.812 | 0.827 |
|  | Hipp_L_2_2 | 0.934 | 0.784 | 0.827 |
|  | Hipp_R_2_2 | 0.927 | 0.812 | 0.861 |
| Basal ganglia(BG) | BG_L_6_1 | 0.996 | 0.597 | 0.600 |
|  | BG_R_6_1 | 0.927 | 0.677 | 0.655 |
|  | BG_L_6_2 | 0.996 | 0.473 | 0.358 |
|  | BG_R_6_2 | 0.914 | 0.473 | 0.358 |
|  | BG_L_6_3 | 0.914 | 0.341 | 0.293 |
|  | BG_R_6_3 | 0.927 | 0.982 | 0.920 |
|  | BG_L_6_4 | 0.927 | 0.440 | 0.358 |
|  | BG_R_6_4 | 0.914 | 0.349 | 0.358 |
|  | BG_L_6_5 | 0.914 | 0.812 | 0.655 |
|  | BG_R_6_5 | 0.870 | 0.853 | 0.972 |
|  | BG_L_6_6 | 0.934 | 0.510 | 0.358 |
|  | BG_R_6_6 | 0.914 | 0.649 | 0.571 |
| Thalamus(Tha) | Tha_L_8_1 | 0.996 | 0.746 | 0.655 |
|  | Tha_R_8_1 | 0.914 | 0.649 | 0.571 |
|  | Tha_L_8_2 | 0.938 | 0.746 | 0.655 |
|  | Tha_R_8_2 | 0.934 | 0.429 | 0.358 |
|  | Tha_L_8_3 | 0.914 | 0.649 | 0.571 |
|  | Tha_R_8_3 | 0.914 | 0.805 | 0.655 |
|  | Tha_L_8_4 | 0.934 | 0.649 | 0.358 |
|  | Tha_R_8_4 | 0.934 | 0.349 | 0.293 |
|  | Tha_L_8_5 | 0.927 | 0.718 | 0.655 |
|  | Tha_R_8_5 | 0.977 | 0.649 | 0.358 |
|  | Tha_L_8_6 | 0.927 | 0.649 | 0.436 |
|  | Tha_R_8_6 | 0.914 | 0.649 | 0.406 |
|  | Tha_L_8_7 | 0.914 | 0.919 | 0.854 |
|  | Tha_R_8_7 | 0.914 | 0.894 | 0.655 |
|  | Tha_L_8_8 | 0.927 | 0.649 | 0.644 |
|  | Tha_R_8_8 | 0.914 | 0.751 | 0.655 |
| Cerebellum(CB) | CB_L_I-IV | 0.934 | 0.982 | 0.988 |
|  | CB_R_I-IV | 0.914 | 0.812 | 0.854 |
|  | CB_L_V | 0.914 | 0.746 | 0.789 |
|  | CB_R_V | 0.927 | 0.904 | 0.920 |
|  | CB_L_VI | 0.956 | 0.706 | 0.655 |
|  | CB_V_VI | 0.368 | 0.706 | 0.655 |
|  | CB_R_VI | 0.927 | 0.894 | 0.925 |
|  | CB_L_Crus_I | 0.927 | 0.883 | 0.854 |
|  | CB_R_Crus_I | 0.934 | 0.812 | 0.854 |
|  | CB_L_Crus_II | 0.927 | 0.773 | 0.708 |
|  | CB_V_Crus_II | 0.927 | 0.992 | 0.917 |
|  | CB_R_Crus_II | 0.934 | 0.718 | 0.655 |
|  | CB_L_VIIb | 0.914 | 0.662 | 0.655 |
|  | CB_V_VIIb | 0.996 | 0.894 | 0.956 |
|  | CB_R_VIIb | 0.956 | 0.784 | 0.698 |
|  | CB_L_VIIIa | 0.927 | 0.791 | 0.714 |
|  | CB_V_VIIIa | 0.938 | 0.904 | 0.987 |
|  | CB_R_VIIIa | 0.928 | 0.982 | 0.854 |
|  | CB_L_VIIIb | 0.927 | 0.933 | 0.925 |
|  | CB_V_VIIIb | 0.927 | 0.894 | 0.956 |
|  | CB_R_VIIIb | 0.927 | 0.822 | 0.682 |
|  | CB_L_IX | 0.927 | 0.894 | 0.988 |
|  | CB_V_IX | 0.945 | 0.711 | 0.714 |
|  | CB_R_IX | 0.934 | 0.892 | 0.975 |
|  | CB_L_X | 0.914 | 0.649 | 0.683 |
|  | CB_V_X | 0.914 | 0.594 | 0.608 |
|  | CB_R_X | 0.914 | 0.746 | 0.854 |
| L, left; R, right; V, vermis.  ^a^ All the nodes are from the Human Brainnetome Atlas.  ^b^ The FDR correction was applied to local topological properties. | | | | |

**Table 4**

Leave-one-subject-out analyses

| Removed participant | *p* (σ) | *p* (γ) |
| --- | --- | --- |
| 1 | 0.037 | 0.031 |
| 2 | 0.046 | 0.034 |
| 3 | 0.037 | 0.031 |
| 4 | 0.010 | 0.006 |
| 5 | 0.050 | 0.037 |
| 6 | 0.043 | 0.031 |
| 7 | 0.040 | 0.034 |
| 8 | 0.046 | 0.037 |
| 9 | 0.050 | 0.037 |
| 10 | 0.031 | 0.028 |
| 11 | 0.020 | 0.014 |
| 12 | 0.040 | 0.031 |
| 13 | 0.046 | 0.037 |
| 14 | 0.040 | 0.037 |
| 15 | 0.008 | 0.006 |
| 16 | 0.046 | 0.037 |
| 17 | 0.046 | 0.037 |
| 18 | 0.018 | 0.020 |
| 19 | 0.046 | 0.037 |
| 20 | 0.040 | 0.034 |
| 21 | 0.040 | 0.034 |

*p* values obtained by excluding one participant at a time and repeating the longitudinal analysis.

**Table 5**

Correlation between global network metrics and mean FD.

| Variable | σ | γ |
| --- | --- | --- |
| FD | 0.195(0.397) | 0.192(0.404) |

Values are presented as r(*p*).

**Table 6**

Changes in nodal topological properties within module 2, 4, 5 and 6 between BL and 6M

| Region | Node ID ^a^ | *p* value ^b^ | | | |
| --- | --- | --- | --- | --- | --- |
|  |  | Betweenness centrality | Degree centrality | Nodal efficiency | Participation coefficient |
| Superior frontal gyrus(SFG) | SFG_L_7_1 | 0.875 | 0.905 | 0.881 | 0.740 |
|  | SFG_R_7_1 | 0.768 | 0.529 | 0.370 | 0.735 |
|  | SFG_L_7_4 | 0.833 | 0.624 | 0.471 | 0.912 |
|  | SFG_R_7_4 | 0.906 | 0.905 | 0.679 | 0.806 |
|  | SFG_L_7_5 | 0.695 | 0.933 | 0.696 | 0.950 |
|  | SFG_R_7_5 | 0.833 | 0.905 | 0.663 | 0.981 |
| Inferior frontal gyrus(IFG) | IFG_R_6_1 | 0.968 | 0.905 | 0.806 | 0.635 |
|  | IFG_R_6_3 | 0.875 | 0.905 | 0.971 | 0.932 |
|  | IFG_R_6_4 | 0.960 | 0.971 | 0.953 | 0.981 |
|  | IFG_L_6_5 | 0.960 | 0.370 | 0.208 | 0.934 |
|  | IFG_R_6_5 | 0.968 | 0.582 | 0.471 | 0.932 |
|  | IFG_L_6_6 | 0.788 | 0.306 | 0.241 | 0.985 |
|  | IFG_R_6_6 | 0.977 | 0.829 | 0.766 | 0.981 |
| Orbital gyrus(OrG) | OrG_R_6_6 | 0.720 | 0.684 | 0.711 | 0.932 |
| Precentral gyrus(PrG) | PrG_L_6_1 | 0.833 | 0.613 | 0.471 | 1.000 |
|  | PrG_R_6_1 | 0.945 | 0.657 | 0.629 | 0.632 |
|  | PrG_L_6_2 | 0.882 | 0.582 | 0.328 | 0.912 |
|  | PrG_R_6_2 | 0.960 | 0.905 | 0.911 | 0.912 |
|  | PrG_L_6_3 | 0.833 | 0.657 | 0.531 | 0.735 |
|  | PrG_R_6_3 | 0.963 | 1.000 | 0.881 | 0.981 |
|  | PrG_L_6_4 | 0.968 | 0.829 | 0.953 | 0.981 |
|  | PrG_R_6_4 | 0.968 | 0.484 | 0.471 | 1.000 |
|  | PrG_L_6_5 | 0.788 | 0.306 | 0.117 | 0.735 |
|  | PrG_R_6_5 | 0.977 | 0.843 | 0.531 | 0.985 |
|  | PrG_L_6_6 | 0.977 | 0.867 | 0.690 | 1.000 |
|  | PrG_R_6_6 | 0.977 | 0.889 | 0.953 | 0.981 |
| Paracentral lobule(PCL) | PCL_L_2_1 | 0.960 | 0.462 | 0.471 | 0.932 |
|  | PCL_R_2_1 | 0.960 | 0.464 | 0.471 | 0.981 |
|  | PCL_L_2_2 | 0.977 | 0.582 | 0.637 | 0.981 |
|  | PCL_R_2_2 | 0.720 | 0.306 | 0.449 | 0.981 |
| Superior temporal gyrus(STG) | STG_L_6_1 | 0.960 | 0.684 | 0.679 | 1.000 |
|  | STG_R_6_1 | 0.695 | 0.696 | 0.723 | 0.912 |
|  | STG_L_6_2 | 0.804 | 0.904 | 0.727 | 0.985 |
|  | STG_R_6_2 | 0.788 | 0.905 | 0.727 | 0.912 |
|  | STG_L_6_3 | 0.968 | 0.816 | 0.531 | 1.000 |
|  | STG_R_6_3 | 0.833 | 0.918 | 0.955 | 0.981 |
|  | STG_L_6_4 | 0.695 | 0.387 | 0.262 | 0.932 |
|  | STG_R_6_4 | 0.833 | 0.746 | 0.746 | 0.981 |
|  | STG_L_6_5 | 0.960 | 0.582 | 0.441 | 0.981 |
|  | STG_R_6_5 | 0.695 | 0.905 | 0.908 | 0.981 |
|  | STG_L_6_6 | 0.841 | 0.764 | 0.766 | 0.981 |
|  | STG_R_6_6 | 0.841 | 1.000 | 0.861 | 0.981 |
| Inferior temporal gyrus(ITG) | ITG_L_7_1 | 0.720 | 0.613 | 0.370 | 0.806 |
|  | ITG_R_7_1 | 0.833 | 0.905 | 0.953 | 0.981 |
|  | ITG_L_7_3 | 0.968 | 0.905 | 0.890 | 0.981 |
|  | ITG_R_7_3 | 0.731 | 0.613 | 0.575 | 0.740 |
|  | ITG_L_7_7 | 0.960 | 0.657 | 0.575 | 0.912 |
|  | ITG_R_7_7 | 0.695 | 0.613 | 0.766 | 0.981 |
| Fusiform gyrus(FuG) | FuG_L_3_1 | 0.875 | 0.529 | 0.449 | 0.932 |
|  | FuG_R_3_1 | 0.960 | 0.766 | 0.876 | 0.981 |
| Parahippocampal gyrus(PhG) | PhG_L_6_1 | 0.695 | 0.306 | 0.243 | 0.740 |
|  | PhG_R_6_1 | 0.960 | 0.496 | 0.359 | 0.735 |
|  | PhG_L_6_2 | 0.874 | 0.613 | 0.584 | 0.735 |
|  | PhG_R_6_2 | 0.720 | 0.904 | 0.881 | 0.981 |
|  | PhG_L_6_3 | 0.720 | 0.878 | 0.997 | 0.934 |
|  | PhG_L_6_4 | 0.695 | 0.477 | 0.370 | 0.981 |
|  | PhG_R_6_4 | 0.977 | 0.905 | 0.523 | 0.806 |
|  | PhG_L_6_5 | 0.960 | 0.306 | 0.328 | 1.000 |
|  | PhG_R_6_5 | 0.960 | 0.387 | 0.244 | 0.735 |
| Posterior superior temporal sulcus(pSTS) | pSTS_L_2_1 | 0.960 | 0.613 | 0.679 | 0.981 |
|  | pSTS_R_2_1 | 0.977 | 0.746 | 0.727 | 0.981 |
|  | pSTS_R_2_2 | 0.833 | 0.613 | 0.531 | 0.981 |
| Superior parietal lobule(SPL) | SPL_R_5_3 | 0.945 | 0.582 | 0.471 | 0.981 |
|  | SPL_L_5_4 | 0.960 | 0.529 | 0.575 | 0.981 |
|  | SPL_R_5_4 | 0.695 | 0.582 | 0.629 | 0.981 |
| Inferior parietal lobule(IPL) | IPL_L_6_3 | 0.960 | 0.613 | 0.679 | 0.932 |
|  | IPL_R_6_4 | 0.859 | 0.793 | 0.637 | 0.981 |
|  | IPL_L_6_6 | 0.960 | 0.805 | 0.600 | 0.981 |
|  | IPL_R_6_6 | 0.841 | 0.878 | 0.908 | 0.981 |
| Postcentral gyrus(PoG) | PoG_L_4_1 | 0.875 | 0.613 | 0.471 | 0.981 |
|  | PoG_R_4_1 | 0.960 | 0.766 | 0.727 | 0.806 |
|  | PoG_L_4_2 | 0.960 | 0.816 | 0.629 | 0.981 |
|  | PoG_R_4_2 | 0.968 | 0.878 | 0.754 | 1.000 |
|  | PoG_L_4_3 | 0.695 | 0.905 | 0.575 | 0.981 |
|  | PoG_R_4_3 | 0.695 | 0.454 | 0.370 | 0.932 |
|  | PoG_L_4_4 | 0.875 | 0.613 | 0.723 | 0.985 |
|  | PoG_R_4_4 | 0.968 | 0.529 | 0.649 | 0.981 |
| Insular gyrus(INS) | INS_L_6_1 | 0.960 | 0.905 | 0.679 | 0.912 |
|  | INS_R_6_1 | 0.720 | 0.700 | 0.911 | 0.981 |
|  | INS_L_6_2 | 0.968 | 0.613 | 0.575 | 0.985 |
|  | INS_R_6_2 | 0.960 | 0.582 | 0.486 | 0.981 |
|  | INS_L_6_3 | 0.960 | 0.582 | 0.471 | 0.740 |
|  | INS_R_6_3 | 0.833 | 0.613 | 0.471 | 0.912 |
|  | INS_L_6_4 | 0.968 | 0.613 | 0.458 | 0.912 |
|  | INS_R_6_4 | 0.960 | 0.816 | 0.908 | 0.985 |
|  | INS_L_6_5 | 0.695 | 0.602 | 0.244 | 0.635 |
|  | INS_R_6_5 | 0.804 | 0.882 | 0.911 | 0.981 |
|  | INS_L_6_6 | 0.695 | 0.306 | 0.140 | 0.237 |
|  | INS_R_6_6 | 0.960 | 0.790 | 0.486 | 0.981 |
| Cingulate gyrus(CG) | CG_L_7_2 | 0.695 | 0.306 | 0.228 | 0.932 |
|  | CG_R_7_2 | 0.977 | 0.582 | 0.471 | 0.981 |
|  | CG_L_7_3 | 0.695 | 0.874 | 0.911 | 0.932 |
|  | CG_R_7_3 | 0.882 | 0.764 | 0.690 | 0.932 |
|  | CG_L_7_5 | 0.960 | 0.951 | 0.738 | 0.932 |
|  | CG_R_7_5 | 0.788 | 0.816 | 0.562 | 0.985 |
|  | CG_L_7_6 | 0.960 | 0.992 | 0.895 | 0.981 |
|  | CG_R_7_6 | 0.875 | 0.905 | 0.813 | 0.981 |
| Amygdala(Amyg) | Amyg_L_2_1 | 0.720 | 0.306 | 0.241 | 0.981 |
|  | Amyg_R_2_1 | 0.841 | 0.613 | 0.452 | 0.912 |
|  | Amyg_L_2_2 | 0.695 | 0.306 | 0.140 | 0.932 |
|  | Amyg_R_2_2 | 0.720 | 0.484 | 0.458 | 0.912 |
| Hippocampus(Hipp) | Hipp_L_2_1 | 0.968 | 0.471 | 0.244 | 0.912 |
|  | Hipp_R_2_1 | 0.968 | 0.641 | 0.531 | 0.981 |
|  | Hipp_L_2_2 | 0.720 | 0.808 | 0.881 | 0.985 |
|  | Hipp_R_2_2 | 0.960 | 0.918 | 0.738 | 0.981 |
| Basal ganglia(BG) | BG_L_6_1 | 0.945 | 0.613 | 0.471 | 0.981 |
|  | BG_R_6_1 | 0.960 | 0.613 | 0.471 | 0.912 |
|  | BG_L_6_2 | 0.960 | 0.582 | 0.244 | 0.932 |
|  | BG_R_6_2 | 0.695 | 0.529 | 0.244 | 0.806 |
|  | BG_L_6_3 | 0.695 | 0.306 | 0.180 | 0.932 |
|  | BG_R_6_3 | 0.841 | 0.971 | 0.849 | 0.981 |
|  | BG_L_6_4 | 0.977 | 0.471 | 0.370 | 0.912 |
|  | BG_R_6_4 | 0.695 | 0.582 | 0.244 | 0.981 |
|  | BG_L_6_5 | 0.960 | 0.790 | 0.679 | 0.981 |
|  | BG_R_6_5 | 0.695 | 0.684 | 0.911 | 0.932 |
|  | BG_L_6_6 | 0.833 | 0.613 | 0.370 | 0.735 |
|  | BG_R_6_6 | 0.695 | 0.613 | 0.471 | 0.981 |
| Thalamus(Tha) | Tha_L_8_1 | 0.968 | 0.684 | 0.471 | 0.981 |
|  | Tha_R_8_1 | 0.804 | 0.387 | 0.359 | 0.964 |
|  | Tha_L_8_2 | 0.720 | 0.867 | 0.728 | 0.912 |
|  | Tha_R_8_2 | 0.859 | 0.582 | 0.262 | 0.912 |
|  | Tha_L_8_3 | 0.875 | 0.835 | 0.433 | 0.912 |
|  | Tha_R_8_3 | 0.945 | 0.805 | 0.330 | 0.912 |
|  | Tha_L_8_4 | 0.875 | 0.684 | 0.244 | 0.932 |
|  | Tha_R_8_4 | 0.695 | 0.529 | 0.188 | 0.632 |
|  | Tha_L_8_5 | 0.968 | 0.816 | 0.471 | 0.981 |
|  | Tha_R_8_5 | 0.960 | 0.684 | 0.244 | 0.632 |
|  | Tha_L_8_6 | 0.804 | 0.764 | 0.348 | 0.735 |
|  | Tha_R_8_6 | 0.720 | 0.825 | 0.370 | 0.735 |
|  | Tha_L_8_7 | 0.955 | 0.905 | 0.629 | 0.981 |
|  | Tha_R_8_7 | 0.960 | 0.829 | 0.649 | 0.981 |
|  | Tha_L_8_8 | 0.977 | 0.766 | 0.441 | 0.932 |
|  | Tha_R_8_8 | 0.695 | 0.613 | 0.471 | 0.981 |
| Cerebellum(CB) | CB_L_I-IV | 0.960 | 0.862 | 0.849 | 0.981 |
|  | CB_R_I-IV | 0.695 | 0.684 | 0.787 | 1.000 |
|  | CB_L_V | 0.833 | 0.684 | 0.727 | 0.912 |
|  | CB_R_V | 0.960 | 1.000 | 0.953 | 0.981 |
|  | CB_L_VI | 0.875 | 0.997 | 0.738 | 0.735 |
|  | CB_V_VI | 0.695 | 1.000 | 0.727 | 0.981 |
|  | CB_R_VI | 0.960 | 0.905 | 0.908 | 0.981 |
|  | CB_L_Crus_I | 0.833 | 0.904 | 0.775 | 0.981 |
|  | CB_R_Crus_I | 0.968 | 0.867 | 0.881 | 0.981 |
|  | CB_L_Crus_II | 0.960 | 0.918 | 0.775 | 0.981 |
|  | CB_V_Crus_II | 0.960 | 0.613 | 0.831 | 0.985 |
|  | CB_L_VIIb | 0.945 | 0.793 | 0.714 | 0.985 |
|  | CB_V_VIIb | 0.960 | 0.582 | 0.637 | 0.981 |
|  | CB_R_VIIb | 0.977 | 0.929 | 0.771 | 0.735 |
|  | CB_L_VIIIa | 0.945 | 0.904 | 0.727 | 0.932 |
|  | CB_V_VIIIa | 0.960 | 0.613 | 0.911 | 0.981 |
|  | CB_R_VIIIa | 0.841 | 0.856 | 0.911 | 0.806 |
|  | CB_L_VIIIb | 0.945 | 0.945 | 0.908 | 1.000 |
|  | CB_V_VIIIb | 0.977 | 0.684 | 0.908 | 0.981 |
|  | CB_R_VIIIb | 0.960 | 0.904 | 0.575 | 0.735 |
|  | CB_L_IX | 0.833 | 0.764 | 0.911 | 0.981 |
|  | CB_V_IX | 0.960 | 0.613 | 0.723 | 0.985 |
|  | CB_R_IX | 0.960 | 0.613 | 0.908 | 0.981 |
|  | CB_L_X | 0.833 | 0.471 | 0.495 | 0.932 |
|  | CB_V_X | 0.841 | 0.306 | 0.370 | 0.735 |
|  | CB_R_X | 0.977 | 0.613 | 0.649 | 0.981 |
| L, left; R, right; V, vermis.  ^a^ All the nodes are from the Human Brainnetome Atlas.  ^b^ The FDR correction was applied to local topological properties. | | | | | |

**Table 7**

Inter-modular connectivity strength in the structural network

|  | BL (mean ± SD) | 6M (mean ± SD) | *p* (uncorr.) | *p* (FDR) |
| --- | --- | --- | --- | --- |
| module1&2 | 1.58±0.20 | 1.59±0.19 | 0.684 | 0.930 |
| module1&3 | 1.67±0.13 | 1.67±0.15 | 0.997 | 0.997 |
| module1&4 | 2.38±0.53 | 2.12±0.69 | 0.092* | 0.439 |
| module1&5 | 2.52±0.40 | 2.44±0.50 | 0.301 | 0.645 |
| module1&6 | 0.72±0.18 | 0.71±0.14 | 0.602 | 0.930 |
| module2&3 | 1.15±0.12 | 1.16±0.12 | 0.841 | 0.970 |
| module2&4 | 0.60±0.11 | 0.57±0.09 | 0.033* | 0.248 |
| module2&5 | 2.09±0.56 | 2.11±0.54 | 0.446 | 0.836 |
| module2&6 | 1.19±0.25 | 1.14±0.21 | 0.020* | 0.248 |
| module3&4 | 0.41±0.34 | 0.55±0.46 | 0.744 | 0.930 |
| module3&5 | 1.96±0.33 | 1.96±0.35 | 0.940 | 0.997 |
| module3&6 | 0.56±0.12 | 0.55±0.09 | 0.117 | 0.439 |
| module4&5 | 0.21±0.19 | 0.19±0.22 | 0.699 | 0.930 |
| module4&6 | 0.83±0.14 | 0.79±0.14 | 0.147 | 0.441 |
| module5&6 | 2.19±0.47 | 2.11±0.57 | 0.280 | 0.645 |

Inter-modular connectivity strength in the structural network before and after MRgFUS treatment is presented, with module pairs exhibiting trend-level decreases at 6 months highlighted (**p* < 0.1). Values represent the average inter-modular connectivity strength.

**Table 8**

Intra-modular connectivity strength in the structural network

|  | BL (mean ± SD) | 6M (mean ± SD) | *p* (uncorr.) | *p* (FDR) |
| --- | --- | --- | --- | --- |
| module1 | 2.79±0.27 | 2.76±0.24 | 0.375 | 0.926 |
| module2 | 2.75±0.26 | 2.73±0.28 | 0.198 | 0.926 |
| module3 | 4.01±0.40 | 4.01±0.43 | 0.969 | 0.969 |
| module4 | 3.18±0.57 | 3.13±0.44 | 0.654 | 0.941 |
| module5 | 5.06±0.70 | 5.00±0.82 | 0.463 | 0.926 |
| module6 | 1.83±0.28 | 1.83±0.29 | 0.784 | 0.941 |

Intra-modular connectivity strength in the structural network before and after MRgFUS treatment is presented. Values represent the average intra-modular connectivity strength.
