# Supplementary material for: Functional network reorganization after magnetic resonance-guided focused ultrasound thalamotomy in tremor-dominant Parkinson's disease patients
Source: Neuroimage Clin. 2026 Sep 5;52:104058. doi: 10.1016/j.nicl.2026.104058 (PMC13573704; doi:10.1016/j.nicl.2026.104058)
Supplement: Supplementary file 3 [file mmc3.docx]

**Table 1**

Inter-modular connectivity in responder

|  | BL (mean ± SD) | 6M (mean ± SD) | *p* (uncorr.) | *p* (FDR) |
| --- | --- | --- | --- | --- |
| module1&2 | 655.70±214.69 | 626.00±127.05 | 0.652 | 0.841 |
| module1&3 | 359.30±98.77 | 401.40±117.87 | 0.272 | 0.745 |
| module1&4 | 72.10±50.60 | 76.60±55.71 | 0.809 | 0.867 |
| module1&5 | 131.90±35.57 | 169.70±80.68 | 0.155 | 0.745 |
| module1&6 | 27.60±40.20 | 15.50±23.95 | 0.206 | 0.745 |
| module2&3 | 602.50±224.74 | 533.20±232.51 | 0.494 | 0.823 |
| module2&4 | 93.70±89.44 | 57.10±47.60 | 0.298 | 0.745 |
| module2&5 | 115.90±46.75 | 132.90±71.93 | 0.586 | 0.841 |
| module2&6 | 45.80±59.62 | 58.90±52.50 | 0.432 | 0.810 |
| module3&4 | 132.10±75.63 | 145.30±108.24 | 0.683 | 0.841 |
| module3&5 | 84.40±39.24 | 100.90±38.20 | 0.364 | 0.780 |
| module3&6 | 8.70±11.66 | 8.70±10.54 | 1.000 | 1.000 |
| module4&5 | 26.30±14.22 | 41.40±16.83 | 0.037* | 0.555 |
| module4&6 | 10.10±10.80 | 4.20±5.43 | 0.136 | 0.745 |
| module5&6 | 10.40±15.79 | 9.30±10.99 | 0.729 | 0.841 |
| Inter-modular functional connectivity before and after MRgFUS treatment in responder is shown. Module pairs with trend-level increases at 6 months are highlighted (**p* < 0.1). | | | | |

**Table 2**

Intra-modular connectivity in responder

|  | BL (mean ± SD) | 6M (mean ± SD) | *p* (uncorr.) | *p* (FDR) |
| --- | --- | --- | --- | --- |
| module1 | 687.60±181.57 | 718.10±127.73 | 0.505 | 0.745 |
| module2 | 1343.20±181.48 | 1365.40±208.30 | 0.745 | 0.745 |
| module3 | 708.10±163.72 | 619.40±77.91 | 0.210 | 0.630 |
| module4 | 137.80±47.30 | 147.10±56.79 | 0.670 | 0.745 |
| module5 | 102.20±27.02 | 122.20±36.14 | 0.190 | 0.630 |
| module6 | 78.60±22.84 | 80.70±15.13 | 0.680 | 0.745 |
| Intra-modular functional connectivity before and after MRgFUS treatment in responder is shown. | | | | |

**Table 3**

Changes in the AUC of nodal topological properties between BL and 6M in responder

| Region | Node ID ^a^ | *p* value ^b^ | | |
| --- | --- | --- | --- | --- |
|  |  | Betweenness centrality | Degree centrality | Nodal efficiency |
| Superior frontal gyrus(SFG) | SFG_L_7_1 | 0.919 | 0.936 | 0.918 |
|  | SFG_R_7_1 | 0.877 | 0.936 | 0.896 |
|  | SFG_L_7_2 | 0.877 | 0.862 | 0.860 |
|  | SFG_R_7_2 | 0.877 | 0.972 | 0.896 |
|  | SFG_L_7_3 | 0.877 | 0.936 | 0.881 |
|  | SFG_R_7_3 | 0.877 | 0.949 | 0.929 |
|  | SFG_L_7_4 | 0.999 | 0.862 | 0.881 |
|  | SFG_R_7_4 | 0.877 | 0.936 | 0.906 |
|  | SFG_L_7_5 | 0.999 | 0.736 | 0.830 |
|  | SFG_R_7_5 | 0.877 | 0.936 | 0.896 |
|  | SFG_L_7_6 | 0.877 | 0.936 | 0.881 |
|  | SFG_R_7_6 | 0.967 | 0.936 | 0.895 |
|  | SFG_L_7_7 | 0.877 | 0.936 | 0.881 |
|  | SFG_R_7_7 | 0.877 | 0.936 | 0.881 |
| Middle frontal gyrus(MFG) | MFG_L_7_1 | 0.877 | 0.975 | 0.935 |
|  | MFG_R_7_1 | 0.888 | 0.975 | 0.943 |
|  | MFG_L_7_2 | 0.877 | 0.862 | 0.860 |
|  | MFG_R_7_2 | 0.999 | 0.832 | 0.860 |
|  | MFG_L_7_3 | 0.877 | 0.936 | 0.881 |
|  | MFG_R_7_3 | 0.877 | 0.936 | 0.907 |
|  | MFG_L_7_4 | 0.877 | 0.975 | 0.898 |
|  | MFG_R_7_4 | 0.877 | 0.936 | 0.881 |
|  | MFG_L_7_5 | 0.887 | 0.862 | 0.860 |
|  | MFG_R_7_5 | 0.877 | 0.936 | 0.881 |
|  | MFG_L_7_6 | 0.907 | 0.927 | 0.881 |
|  | MFG_R_7_6 | 0.880 | 0.975 | 0.918 |
|  | MFG_L_7_7 | 0.877 | 0.936 | 0.881 |
|  | MFG_R_7_7 | 0.919 | 0.936 | 0.881 |
| Inferior frontal gyrus(IFG) | IFG_L_6_1 | 0.999 | 0.987 | 0.935 |
|  | IFG_R_6_1 | 0.958 | 0.975 | 0.996 |
|  | IFG_L_6_2 | 0.877 | 0.963 | 0.896 |
|  | IFG_R_6_2 | 0.877 | 0.832 | 0.762 |
|  | IFG_L_6_3 | 0.877 | 0.900 | 0.860 |
|  | IFG_R_6_3 | 0.877 | 0.736 | 0.718 |
|  | IFG_L_6_4 | 0.877 | 0.832 | 0.860 |
|  | IFG_R_6_4 | 0.877 | 0.862 | 0.881 |
|  | IFG_L_6_5 | 0.877 | 0.832 | 0.762 |
|  | IFG_R_6_5 | 0.951 | 0.862 | 0.862 |
|  | IFG_L_6_6 | 0.999 | 0.762 | 0.715 |
|  | IFG_R_6_6 | 0.877 | 0.936 | 0.881 |
| Orbital gyrus(OrG) | OrG_L_6_1 | 0.877 | 0.975 | 0.896 |
|  | OrG_R_6_1 | 0.877 | 0.936 | 0.896 |
|  | OrG_L_6_2 | 0.984 | 0.936 | 0.881 |
|  | OrG_R_6_2 | 0.965 | 0.943 | 0.881 |
|  | OrG_L_6_3 | 0.919 | 0.862 | 0.831 |
|  | OrG_R_6_3 | 0.877 | 0.900 | 0.895 |
|  | OrG_L_6_4 | 0.888 | 0.969 | 0.881 |
|  | OrG_R_6_4 | 0.877 | 0.936 | 0.921 |
|  | OrG_L_6_5 | 0.877 | 0.936 | 0.881 |
|  | OrG_R_6_5 | 0.877 | 0.985 | 0.907 |
|  | OrG_L_6_6 | 0.877 | 0.877 | 0.862 |
|  | OrG_R_6_6 | 0.877 | 0.832 | 0.770 |
| Precentral gyrus(PrG) | PrG_L_6_1 | 0.877 | 0.936 | 0.896 |
|  | PrG_R_6_1 | 0.877 | 0.943 | 0.896 |
|  | PrG_L_6_2 | 0.886 | 0.975 | 0.896 |
|  | PrG_R_6_2 | 0.999 | 0.936 | 0.896 |
|  | PrG_L_6_3 | 0.956 | 0.969 | 0.918 |
|  | PrG_R_6_3 | 0.877 | 0.936 | 0.896 |
|  | PrG_L_6_4 | 0.970 | 0.687 | 0.596 |
|  | PrG_R_6_4 | 0.877 | 0.736 | 0.762 |
|  | PrG_L_6_5 | 0.877 | 0.687 | 0.586 |
|  | PrG_R_6_5 | 0.888 | 0.936 | 0.881 |
|  | PrG_L_6_6 | 0.877 | 0.832 | 0.770 |
|  | PrG_R_6_6 | 0.919 | 0.975 | 0.953 |
| Paracentral lobule(PCL) | PCL_L_2_1 | 0.967 | 0.687 | 0.596 |
|  | PCL_R_2_1 | 0.877 | 0.563 | 0.586 |
|  | PCL_L_2_2 | 0.877 | 0.736 | 0.715 |
|  | PCL_R_2_2 | 0.877 | 0.687 | 0.715 |
| Superior temporal gyrus(STG) | STG_L_6_1 | 0.877 | 0.832 | 0.770 |
|  | STG_R_6_1 | 0.877 | 0.936 | 0.881 |
|  | STG_L_6_2 | 0.999 | 0.936 | 0.918 |
|  | STG_R_6_2 | 0.919 | 0.862 | 0.881 |
|  | STG_L_6_3 | 0.958 | 0.972 | 0.896 |
|  | STG_R_6_3 | 0.877 | 0.687 | 0.715 |
|  | STG_L_6_4 | 0.877 | 0.936 | 0.896 |
|  | STG_R_6_4 | 0.877 | 0.936 | 0.881 |
|  | STG_L_6_5 | 0.877 | 0.936 | 0.881 |
|  | STG_R_6_5 | 0.877 | 0.900 | 0.881 |
|  | STG_L_6_6 | 0.877 | 0.975 | 0.896 |
|  | STG_R_6_6 | 0.877 | 0.975 | 0.906 |
| Middle temporal gyrus(MTG) | MTG_L_4_1 | 0.979 | 0.943 | 0.896 |
|  | MTG_R_4_1 | 0.877 | 0.936 | 0.881 |
|  | MTG_L_4_2 | 0.877 | 0.832 | 0.860 |
|  | MTG_R_4_2 | 0.877 | 0.862 | 0.860 |
|  | MTG_L_4_3 | 0.877 | 0.877 | 0.881 |
|  | MTG_R_4_3 | 0.877 | 0.687 | 0.586 |
|  | MTG_L_4_4 | 0.877 | 0.936 | 0.898 |
|  | MTG_R_4_4 | 0.877 | 0.936 | 0.881 |
| Inferior temporal gyrus(ITG) | ITG_L_7_1 | 0.877 | 0.582 | 0.586 |
|  | ITG_R_7_1 | 0.877 | 0.987 | 0.921 |
|  | ITG_L_7_2 | 0.877 | 0.762 | 0.762 |
|  | ITG_R_7_2 | 0.877 | 0.972 | 0.942 |
|  | ITG_L_7_3 | 0.877 | 0.969 | 0.953 |
|  | ITG_R_7_3 | 0.877 | 0.936 | 0.881 |
|  | ITG_L_7_4 | 0.967 | 0.862 | 0.860 |
|  | ITG_R_7_4 | 0.877 | 0.891 | 0.881 |
|  | ITG_L_7_5 | 0.877 | 0.975 | 0.983 |
|  | ITG_R_7_5 | 0.877 | 0.563 | 0.586 |
|  | ITG_L_7_6 | 0.877 | 0.762 | 0.762 |
|  | ITG_R_7_6 | 0.877 | 0.862 | 0.881 |
|  | ITG_L_7_7 | 0.888 | 0.621 | 0.586 |
|  | ITG_R_7_7 | 0.877 | 0.936 | 0.929 |
| Fusiform gyrus(FuG) | FuG_L_3_1 | 0.877 | 0.936 | 0.881 |
|  | FuG_R_3_1 | 0.877 | 0.975 | 0.943 |
|  | FuG_L_3_2 | 0.877 | 0.936 | 0.881 |
|  | FuG_R_3_2 | 0.877 | 0.832 | 0.860 |
|  | FuG_L_3_3 | 0.877 | 0.862 | 0.860 |
|  | FuG_R_3_3 | 0.877 | 0.975 | 0.922 |
| Parahippocampal gyrus(PhG) | PhG_L_6_1 | 0.877 | 0.714 | 0.586 |
|  | PhG_R_6_1 | 0.888 | 0.736 | 0.586 |
|  | PhG_L_6_2 | 0.877 | 0.936 | 0.881 |
|  | PhG_R_6_2 | 0.877 | 0.832 | 0.860 |
|  | PhG_L_6_3 | 0.877 | 0.936 | 0.881 |
|  | PhG_R_6_3 | 0.919 | 0.950 | 0.922 |
|  | PhG_L_6_4 | 0.877 | 0.936 | 0.881 |
|  | PhG_R_6_4 | 0.877 | 0.969 | 0.922 |
|  | PhG_L_6_5 | 0.877 | 0.762 | 0.770 |
|  | PhG_R_6_5 | 0.877 | 0.687 | 0.586 |
|  | PhG_L_6_6 | 0.877 | 0.969 | 0.896 |
|  | PhG_R_6_6 | 0.877 | 0.975 | 0.921 |
| Posterior superior temporal sulcus(pSTS) | pSTS_L_2_1 | 0.901 | 0.936 | 0.881 |
|  | pSTS_R_2_1 | 0.877 | 0.936 | 0.881 |
|  | pSTS_L_2_2 | 0.969 | 0.950 | 0.929 |
|  | pSTS_R_2_2 | 0.877 | 0.862 | 0.881 |
| Superior parietal lobule(SPL) | SPL_L_5_1 | 0.877 | 0.969 | 0.957 |
|  | SPL_R_5_1 | 0.877 | 0.832 | 0.860 |
|  | SPL_L_5_2 | 0.877 | 0.936 | 0.896 |
|  | SPL_R_5_2 | 0.877 | 0.943 | 0.935 |
|  | SPL_L_5_3 | 0.877 | 0.936 | 0.881 |
|  | SPL_R_5_3 | 0.877 | 0.936 | 0.896 |
|  | SPL_L_5_4 | 0.877 | 0.832 | 0.860 |
|  | SPL_R_5_4 | 0.919 | 0.862 | 0.860 |
|  | SPL_L_5_5 | 0.877 | 0.862 | 0.860 |
|  | SPL_R_5_5 | 0.958 | 0.975 | 0.907 |
| Inferior parietal lobule(IPL) | IPL_L_6_1 | 0.877 | 0.975 | 0.953 |
|  | IPL_R_6_1 | 0.877 | 0.862 | 0.881 |
|  | IPL_L_6_2 | 0.888 | 0.765 | 0.762 |
|  | IPL_R_6_2 | 0.919 | 0.862 | 0.860 |
|  | IPL_L_6_3 | 0.877 | 0.958 | 0.881 |
|  | IPL_R_6_3 | 0.877 | 0.862 | 0.881 |
|  | IPL_L_6_4 | 0.979 | 0.736 | 0.762 |
|  | IPL_R_6_4 | 0.877 | 0.972 | 0.944 |
|  | IPL_L_6_5 | 0.877 | 0.943 | 0.944 |
|  | IPL_R_6_5 | 0.877 | 0.862 | 0.881 |
|  | IPL_L_6_6 | 0.877 | 0.975 | 0.896 |
|  | IPL_R_6_6 | 0.888 | 0.862 | 0.881 |
| Precuneus(Pcun) | PCun_L_4_1 | 0.877 | 0.972 | 0.953 |
|  | PCun_R_4_1 | 0.877 | 0.972 | 0.935 |
|  | PCun_L_4_2 | 0.877 | 0.582 | 0.586 |
|  | PCun_R_4_2 | 0.877 | 0.736 | 0.762 |
|  | PCun_L_4_3 | 0.877 | 0.972 | 0.993 |
|  | PCun_R_4_3 | 0.877 | 0.936 | 0.881 |
|  | PCun_L_4_4 | 0.919 | 0.936 | 0.881 |
|  | PCun_R_4_4 | 0.919 | 0.936 | 0.881 |
| Postcentral gyrus(PoG) | PoG_L_4_1 | 0.877 | 0.862 | 0.862 |
|  | PoG_R_4_1 | 0.999 | 0.950 | 0.907 |
|  | PoG_L_4_2 | 0.877 | 0.936 | 0.881 |
|  | PoG_R_4_2 | 0.887 | 0.936 | 0.881 |
|  | PoG_L_4_3 | 0.877 | 0.972 | 0.896 |
|  | PoG_R_4_3 | 0.877 | 0.891 | 0.881 |
|  | PoG_L_4_4 | 0.877 | 0.832 | 0.860 |
|  | PoG_R_4_4 | 0.877 | 0.762 | 0.770 |
| Insular gyrus(INS) | INS_L_6_1 | 0.877 | 0.985 | 0.921 |
|  | INS_R_6_1 | 0.877 | 0.832 | 0.862 |
|  | INS_L_6_2 | 0.877 | 0.936 | 0.881 |
|  | INS_R_6_2 | 0.958 | 0.943 | 0.896 |
|  | INS_L_6_3 | 0.877 | 0.877 | 0.860 |
|  | INS_R_6_3 | 0.877 | 0.936 | 0.881 |
|  | INS_L_6_4 | 0.877 | 0.969 | 0.906 |
|  | INS_R_6_4 | 0.877 | 0.862 | 0.860 |
|  | INS_L_6_5 | 0.877 | 0.862 | 0.860 |
|  | INS_R_6_5 | 0.956 | 0.936 | 0.896 |
|  | INS_L_6_6 | 0.877 | 0.771 | 0.762 |
|  | INS_R_6_6 | 0.965 | 0.936 | 0.881 |
| Cingulate gyrus(CG) | CG_L_7_1 | 0.958 | 0.975 | 0.935 |
|  | CG_R_7_1 | 0.877 | 0.927 | 0.881 |
|  | CG_L_7_2 | 0.877 | 0.936 | 0.881 |
|  | CG_R_7_2 | 0.877 | 0.978 | 0.953 |
|  | CG_L_7_3 | 0.877 | 0.972 | 0.922 |
|  | CG_R_7_3 | 0.877 | 0.975 | 0.921 |
|  | CG_L_7_4 | 0.933 | 0.714 | 0.762 |
|  | CG_R_7_4 | 0.877 | 0.987 | 0.896 |
|  | CG_L_7_5 | 0.919 | 0.936 | 0.896 |
|  | CG_R_7_5 | 0.901 | 0.936 | 0.895 |
|  | CG_L_7_6 | 0.877 | 0.950 | 0.906 |
|  | CG_R_7_6 | 0.877 | 0.975 | 0.944 |
|  | CG_L_7_7 | 0.877 | 0.936 | 0.881 |
|  | CG_R_7_7 | 0.877 | 0.950 | 0.896 |
| MedioVentral occipital cortex(MVOcC) | MVOcC_L_5_1 | 0.956 | 0.936 | 0.896 |
|  | MVOcC_R_5_1 | 0.999 | 0.862 | 0.881 |
|  | MVOcC_L_5_2 | 0.958 | 0.832 | 0.862 |
|  | MVOcC_R_5_2 | 0.958 | 0.832 | 0.860 |
|  | MVOcC_L_5_3 | 0.877 | 0.862 | 0.881 |
|  | MVOcC_R_5_3 | 0.951 | 0.832 | 0.860 |
|  | MVOcC_L_5_4 | 0.919 | 0.736 | 0.860 |
|  | MVOcC_R_5_4 | 0.877 | 0.832 | 0.860 |
|  | MVOcC_L_5_5 | 0.877 | 0.936 | 0.896 |
|  | MVOcC_R_5_5 | 0.877 | 0.736 | 0.773 |
| Lateral occipital cortex(LOcC) | LOcC_L_4_1 | 0.923 | 0.975 | 0.918 |
|  | LOcC_R_4_1 | 0.888 | 0.969 | 0.929 |
|  | LOcC_L_4_2 | 0.877 | 0.975 | 0.922 |
|  | LOcC_R_4_2 | 0.958 | 0.936 | 0.896 |
|  | LOcC_L_4_3 | 0.877 | 0.972 | 0.953 |
|  | LOcC_R_4_3 | 0.877 | 0.950 | 0.929 |
|  | LOcC_L_4_4 | 0.888 | 0.936 | 0.881 |
|  | LOcC_R_4_4 | 0.877 | 0.958 | 0.918 |
|  | LOcC_L_2_1 | 0.877 | 0.936 | 0.881 |
|  | LOcC_R_2_1 | 0.877 | 0.969 | 0.896 |
|  | LOcC_L_2_2 | 0.877 | 0.936 | 0.881 |
|  | LOcC_R_2_2 | 0.877 | 0.959 | 0.929 |
| Amygdala(Amyg) | Amyg_L_2_1 | 0.877 | 0.832 | 0.762 |
|  | Amyg_R_2_1 | 0.877 | 0.903 | 0.860 |
|  | Amyg_L_2_2 | 0.877 | 0.687 | 0.586 |
|  | Amyg_R_2_2 | 0.877 | 0.714 | 0.596 |
| Hippocampus(Hipp) | Hipp_L_2_1 | 0.877 | 0.832 | 0.763 |
|  | Hipp_R_2_1 | 0.888 | 0.936 | 0.881 |
|  | Hipp_L_2_2 | 0.877 | 0.891 | 0.881 |
|  | Hipp_R_2_2 | 0.877 | 0.950 | 0.935 |
| Basal ganglia(BG) | BG_L_6_1 | 0.901 | 0.862 | 0.860 |
|  | BG_R_6_1 | 0.877 | 0.941 | 0.881 |
|  | BG_L_6_2 | 0.965 | 0.936 | 0.830 |
|  | BG_R_6_2 | 0.999 | 0.975 | 0.881 |
|  | BG_L_6_3 | 0.877 | 0.687 | 0.596 |
|  | BG_R_6_3 | 0.901 | 0.975 | 0.896 |
|  | BG_L_6_4 | 0.965 | 0.936 | 0.860 |
|  | BG_R_6_4 | 0.877 | 0.736 | 0.586 |
|  | BG_L_6_5 | 0.877 | 0.975 | 0.881 |
|  | BG_R_6_5 | 0.877 | 0.972 | 0.896 |
|  | BG_L_6_6 | 0.877 | 0.975 | 0.896 |
|  | BG_R_6_6 | 0.877 | 0.975 | 0.895 |
| Thalamus(Tha) | Tha_L_8_1 | 1.000 | 0.949 | 0.881 |
|  | Tha_R_8_1 | 0.877 | 0.936 | 0.907 |
|  | Tha_L_8_2 | 0.920 | 0.975 | 0.896 |
|  | Tha_R_8_2 | 0.877 | 0.950 | 0.881 |
|  | Tha_L_8_3 | 0.877 | 0.936 | 0.881 |
|  | Tha_R_8_3 | 0.877 | 0.936 | 0.934 |
|  | Tha_L_8_4 | 0.877 | 0.862 | 0.586 |
|  | Tha_R_8_4 | 0.923 | 0.862 | 0.830 |
|  | Tha_L_8_5 | 0.877 | 0.936 | 0.881 |
|  | Tha_R_8_5 | 0.877 | 0.950 | 0.860 |
|  | Tha_L_8_6 | 0.877 | 0.862 | 0.836 |
|  | Tha_R_8_6 | 0.877 | 0.736 | 0.596 |
|  | Tha_L_8_7 | 0.888 | 0.969 | 0.881 |
|  | Tha_R_8_7 | 0.877 | 0.980 | 0.881 |
|  | Tha_L_8_8 | 0.877 | 0.975 | 0.896 |
|  | Tha_R_8_8 | 0.877 | 0.862 | 0.881 |
| Cerebellum(CB) | CB_L_I-IV | 0.877 | 0.877 | 0.881 |
|  | CB_R_I-IV | 0.888 | 0.936 | 0.906 |
|  | CB_L_V | 0.877 | 0.936 | 0.881 |
|  | CB_R_V | 0.877 | 0.993 | 0.944 |
|  | CB_L_VI | 0.877 | 0.862 | 0.860 |
|  | CB_V_VI | 0.877 | 0.877 | 0.881 |
|  | CB_R_VI | 0.951 | 0.975 | 0.943 |
|  | CB_L_Crus_I | 0.888 | 0.936 | 0.881 |
|  | CB_R_Crus_I | 0.919 | 0.950 | 0.896 |
|  | CB_L_Crus_II | 0.999 | 0.975 | 0.935 |
|  | CB_V_Crus_II | 0.877 | 0.936 | 0.881 |
|  | CB_R_Crus_II | 0.877 | 0.936 | 0.881 |
|  | CB_L_VIIb | 0.956 | 0.975 | 0.995 |
|  | CB_V_VIIb | 0.901 | 0.936 | 0.898 |
|  | CB_R_VIIb | 0.877 | 0.908 | 0.881 |
|  | CB_L_VIIIa | 0.888 | 0.972 | 0.922 |
|  | CB_V_VIIIa | 0.877 | 0.943 | 0.896 |
|  | CB_R_VIIIa | 0.877 | 0.936 | 0.881 |
|  | CB_L_VIIIb | 0.877 | 0.950 | 0.935 |
|  | CB_V_VIIIb | 0.877 | 0.987 | 0.953 |
|  | CB_R_VIIIb | 0.877 | 0.832 | 0.860 |
|  | CB_L_IX | 0.877 | 0.832 | 0.881 |
|  | CB_V_IX | 0.984 | 0.950 | 0.881 |
|  | CB_R_IX | 0.877 | 0.936 | 0.896 |
|  | CB_L_X | 0.877 | 0.936 | 0.881 |
|  | CB_V_X | 0.877 | 0.762 | 0.762 |
|  | CB_R_X | 0.877 | 0.832 | 0.881 |
| L, left; R, right; V, vermis.  ^a^ All the nodes are from the Human Brainnetome Atlas.  ^b^ The FDR correction was applied to local topological properties. | | | | |

**Table 4**

Changes in nodal topological properties within module 4 and 5 between BL and 6M in responder

| Region | Node ID ^a^ | *p* value ^b^ | | | |
| --- | --- | --- | --- | --- | --- |
|  |  | Betweenness centrality | Degree centrality | Nodal efficiency | Participation coefficient |
| Superior temporal gyrus(STG) | STG_L_6_1 | 0.874 | 0.987 | 0.928 | 0.979 |
|  | STG_R_6_1 | 0.784 | 0.987 | 0.928 | 0.921 |
| Inferior temporal gyrus(ITG) | ITG_L_7_1 | 0.784 | 0.571 | 0.187 | 0.611 |
|  | ITG_R_7_1 | 0.821 | 0.987 | 0.928 | 0.999 |
|  | ITG_L_7_3 | 0.996 | 0.987 | 0.928 | 0.739 |
|  | ITG_R_7_3 | 0.823 | 0.987 | 0.928 | 0.480 |
|  | ITG_L_7_7 | 0.821 | 0.455 | 0.179 | 0.739 |
|  | ITG_R_7_7 | 0.784 | 0.792 | 0.928 | 0.480 |
| Fusiform gyrus(FuG) | FuG_L_3_1 | 0.784 | 0.571 | 0.461 | 0.921 |
|  | FuG_R_3_1 | 0.874 | 0.987 | 0.928 | 0.999 |
| Parahippocampal gyrus(PhG) | PhG_L_6_1 | 0.784 | 0.122 | 0.179 | 0.480 |
|  | PhG_R_6_1 | 0.996 | 0.455 | 0.179 | 0.480 |
|  | PhG_L_6_2 | 0.874 | 0.987 | 0.928 | 0.611 |
|  | PhG_R_6_2 | 0.784 | 0.987 | 0.928 | 0.999 |
|  | PhG_L_6_3 | 0.996 | 0.571 | 0.928 | 0.979 |
|  | PhG_L_6_4 | 0.784 | 0.792 | 0.890 | 0.921 |
|  | PhG_R_6_4 | 0.922 | 0.987 | 0.978 | 0.979 |
|  | PhG_L_6_5 | 0.996 | 0.792 | 0.880 | 0.999 |
|  | PhG_R_6_5 | 0.996 | 0.455 | 0.179 | 0.666 |
| Hippocampus(Hipp) | Hipp_L_2_1 | 0.996 | 0.792 | 0.587 | 0.979 |
|  | Hipp_R_2_1 | 0.784 | 0.987 | 0.928 | 0.979 |
|  | Hipp_L_2_2 | 0.784 | 0.987 | 0.928 | 0.979 |
|  | Hipp_R_2_2 | 0.898 | 0.987 | 0.978 | 0.999 |
| Cerebellum(CB) | CB_L_I-IV | 0.996 | 0.987 | 0.978 | 0.979 |
|  | CB_R_I-IV | 0.784 | 0.987 | 0.978 | 0.999 |
|  | CB_L_V | 0.996 | 0.987 | 0.928 | 0.979 |
|  | CB_R_V | 0.996 | 0.987 | 0.928 | 0.999 |
|  | CB_L_VI | 0.784 | 0.987 | 0.928 | 0.489 |
|  | CB_V_VI | 0.784 | 0.987 | 0.928 | 0.979 |
|  | CB_R_VI | 0.996 | 0.987 | 0.978 | 0.979 |
|  | CB_L_Crus_I | 0.821 | 0.987 | 0.928 | 0.979 |
|  | CB_R_Crus_I | 0.996 | 0.988 | 0.928 | 0.999 |
|  | CB_L_Crus_II | 0.784 | 0.987 | 0.928 | 0.979 |
|  | CB_V_Crus_II | 0.874 | 0.988 | 0.928 | 0.999 |
|  | CB_L_VIIb | 0.821 | 0.987 | 0.978 | 0.921 |
|  | CB_V_VIIb | 0.963 | 0.987 | 0.928 | 0.979 |
|  | CB_R_VIIb | 0.898 | 0.987 | 0.928 | 0.739 |
|  | CB_L_VIIIa | 0.996 | 0.987 | 0.928 | 0.979 |
|  | CB_V_VIIIa | 0.821 | 0.987 | 0.928 | 0.739 |
|  | CB_R_VIIIa | 0.784 | 0.987 | 0.928 | 0.739 |
|  | CB_L_VIIIb | 0.784 | 0.987 | 0.928 | 0.979 |
|  | CB_V_VIIIb | 0.898 | 0.987 | 0.928 | 0.979 |
|  | CB_R_VIIIb | 0.874 | 0.680 | 0.890 | 0.480 |
|  | CB_L_IX | 0.784 | 0.987 | 0.928 | 0.979 |
|  | CB_V_IX | 0.963 | 0.987 | 0.928 | 0.921 |
|  | CB_R_IX | 0.784 | 0.816 | 0.928 | 0.999 |
|  | CB_L_X | 0.898 | 0.987 | 0.928 | 0.979 |
|  | CB_V_X | 0.821 | 0.571 | 0.750 | 0.904 |
|  | CB_R_X | 0.821 | 0.987 | 0.928 | 0.979 |
| L, left; R, right; V, vermis.  ^a^ All the nodes are from the Human Brainnetome Atlas.  ^b^ The FDR correction was applied to local topological properties. | | | | | |
