# Supplementary material for: Functional network reorganization after magnetic resonance-guided focused ultrasound thalamotomy in tremor-dominant Parkinson's disease patients
Source: Neuroimage Clin. 2026 Sep 5;52:104058. doi: 10.1016/j.nicl.2026.104058 (PMC13573704; doi:10.1016/j.nicl.2026.104058)
Supplement: Supplementary file 4 [file mmc4.docx]

**Fig. 1.** Alterations in the AUC of global topological properties between BL and 6M in relapser.

**Table 1**

Inter-modular connectivity in relapser

|  | BL (mean ± SD) | 6M (mean ± SD) | *p* (uncorr.) | *p* (FDR) |
| --- | --- | --- | --- | --- |
| module1&2 | 581.44±191.89 | 649.56±155.33 | 0.317 | 0.557 |
| module1&3 | 412.00±105.75 | 309.00±167.68 | 0.115 | 0.557 |
| module1&4 | 91.33±58.15 | 88.33±58.69 | 0.900 | 0.964 |
| module1&5 | 145.11±43.48 | 127.33±56.95 | 0.452 | 0.569 |
| module1&6 | 32.33±22.64 | 46.89±44.04 | 0.345 | 0.557 |
| module2&3 | 506.00±201.18 | 457.67±98.86 | 0.493 | 0.569 |
| module2&4 | 62.33±39.59 | 77.22±50.58 | 0.371 | 0.557 |
| module2&5 | 108.78±34.30 | 169.22±130.96 | 0.219 | 0.557 |
| module2&6 | 44.56±26.44 | 84.56±42.97 | 0.082* | 0.557 |
| module3&4 | 168.89±94.52 | 210.33±203.26 | 0.471 | 0.569 |
| module3&5 | 114.00±48.75 | 97.22±56.73 | 0.250 | 0.557 |
| module3&6 | 22.67±21.42 | 22.44±15.49 | 0.977 | 0.977 |
| module4&5 | 45.78±26.16 | 62.89±59.55 | 0.348 | 0.557 |
| module4&6 | 16.56±26.19 | 19.56±29.62 | 0.137 | 0.557 |
| module5&6 | 12.22±12.70 | 17.11±15.24 | 0.306 | 0.557 |
| Inter-modular functional connectivity before and after MRgFUS treatment in relapser is shown. Module pairs with trend-level increases at 6 months are highlighted (**p* < 0.1). | | | | |

**Table 2**

Intra-modular connectivity in relapser

|  | BL (mean ± SD) | 6M (mean ± SD) | *p* (uncorr.) | *p* (FDR) |
| --- | --- | --- | --- | --- |
| module1 | 728.11±135.37 | 617.33±57.97 | 0.018* | 0.090 |
| module2 | 1313.33±180.03 | 1288.22±264.72 | 0.759 | 0.770 |
| module3 | 713.56±114.59 | 728.89±181.08 | 0.770 | 0.770 |
| module4 | 133.33±22.36 | 161.89±43.08 | 0.030* | 0.090 |
| module5 | 103.89±21.76 | 113.56±30.28 | 0.250 | 0.500 |
| module6 | 77.78±21.48 | 84.78±19.96 | 0.390 | 0.585 |
| Intra-modular functional connectivity before and after MRgFUS treatment in relapser is shown. Modules with trend-level changes at 6 months are highlighted (**p* < 0.1). | | | | |

**Table 3**

Changes in the AUC of nodal topological properties between BL and 6M in relapser

| Region | Node ID ^a^ | *p* value ^b^ | | |
| --- | --- | --- | --- | --- |
|  |  | Betweenness centrality | Degree centrality | Nodal efficiency |
| Superior frontal gyrus(SFG) | SFG_L_7_1 | 1.000 | 0.886 | 0.907 |
|  | SFG_R_7_1 | 1.000 | 0.842 | 0.842 |
|  | SFG_L_7_2 | 1.000 | 0.842 | 0.852 |
|  | SFG_R_7_2 | 1.000 | 0.855 | 0.852 |
|  | SFG_L_7_3 | 0.870 | 0.970 | 0.983 |
|  | SFG_R_7_3 | 0.870 | 0.886 | 0.873 |
|  | SFG_L_7_4 | 0.870 | 0.626 | 0.608 |
|  | SFG_R_7_4 | 0.870 | 0.842 | 0.802 |
|  | SFG_L_7_5 | 0.870 | 0.871 | 0.784 |
|  | SFG_R_7_5 | 1.000 | 0.879 | 0.852 |
|  | SFG_L_7_6 | 1.000 | 0.886 | 0.907 |
|  | SFG_R_7_6 | 1.000 | 0.833 | 0.842 |
|  | SFG_L_7_7 | 1.000 | 0.995 | 0.983 |
|  | SFG_R_7_7 | 1.000 | 0.991 | 0.993 |
| Middle frontal gyrus(MFG) | MFG_L_7_1 | 1.000 | 0.899 | 0.907 |
|  | MFG_R_7_1 | 1.000 | 0.899 | 0.950 |
|  | MFG_L_7_2 | 1.000 | 0.655 | 0.700 |
|  | MFG_R_7_2 | 1.000 | 0.755 | 0.782 |
|  | MFG_L_7_3 | 1.000 | 0.886 | 0.873 |
|  | MFG_R_7_3 | 1.000 | 0.963 | 0.983 |
|  | MFG_L_7_4 | 1.000 | 0.884 | 0.851 |
|  | MFG_R_7_4 | 1.000 | 0.704 | 0.700 |
|  | MFG_L_7_5 | 1.000 | 0.871 | 0.897 |
|  | MFG_R_7_5 | 0.870 | 0.626 | 0.608 |
|  | MFG_L_7_6 | 1.000 | 0.842 | 0.852 |
|  | MFG_R_7_6 | 1.000 | 0.833 | 0.816 |
|  | MFG_L_7_7 | 1.000 | 0.991 | 0.983 |
|  | MFG_R_7_7 | 0.983 | 0.865 | 0.852 |
| Inferior frontal gyrus(IFG) | IFG_L_6_1 | 0.870 | 0.655 | 0.608 |
|  | IFG_R_6_1 | 1.000 | 0.929 | 0.907 |
|  | IFG_L_6_2 | 1.000 | 0.626 | 0.608 |
|  | IFG_R_6_2 | 0.963 | 0.757 | 0.782 |
|  | IFG_L_6_3 | 1.000 | 0.894 | 0.943 |
|  | IFG_R_6_3 | 1.000 | 0.842 | 0.852 |
|  | IFG_L_6_4 | 1.000 | 0.959 | 0.950 |
|  | IFG_R_6_4 | 1.000 | 0.886 | 0.873 |
|  | IFG_L_6_5 | 1.000 | 0.842 | 0.792 |
|  | IFG_R_6_5 | 1.000 | 0.894 | 0.907 |
|  | IFG_L_6_6 | 1.000 | 0.925 | 0.976 |
|  | IFG_R_6_6 | 1.000 | 0.856 | 0.852 |
| Orbital gyrus(OrG) | OrG_L_6_1 | 1.000 | 0.991 | 0.983 |
|  | OrG_R_6_1 | 1.000 | 0.886 | 0.852 |
|  | OrG_L_6_2 | 1.000 | 0.962 | 0.950 |
|  | OrG_R_6_2 | 1.000 | 0.942 | 0.907 |
|  | OrG_L_6_3 | 1.000 | 0.626 | 0.608 |
|  | OrG_R_6_3 | 1.000 | 0.955 | 0.993 |
|  | OrG_L_6_4 | 1.000 | 0.991 | 0.993 |
|  | OrG_R_6_4 | 1.000 | 0.864 | 0.852 |
|  | OrG_L_6_5 | 1.000 | 0.842 | 0.852 |
|  | OrG_R_6_5 | 1.000 | 0.894 | 0.983 |
|  | OrG_L_6_6 | 1.000 | 0.991 | 0.993 |
|  | OrG_R_6_6 | 1.000 | 0.886 | 0.852 |
| Precentral gyrus(PrG) | PrG_L_6_1 | 1.000 | 0.962 | 0.950 |
|  | PrG_R_6_1 | 1.000 | 0.789 | 0.784 |
|  | PrG_L_6_2 | 1.000 | 0.886 | 0.852 |
|  | PrG_R_6_2 | 1.000 | 0.890 | 0.887 |
|  | PrG_L_6_3 | 0.870 | 0.782 | 0.765 |
|  | PrG_R_6_3 | 1.000 | 0.864 | 0.852 |
|  | PrG_L_6_4 | 1.000 | 0.782 | 0.782 |
|  | PrG_R_6_4 | 1.000 | 0.991 | 0.993 |
|  | PrG_L_6_5 | 1.000 | 0.871 | 0.782 |
|  | PrG_R_6_5 | 1.000 | 0.962 | 0.993 |
|  | PrG_L_6_6 | 1.000 | 0.842 | 0.852 |
|  | PrG_R_6_6 | 1.000 | 0.842 | 0.852 |
| Paracentral lobule(PCL) | PCL_L_2_1 | 1.000 | 0.954 | 0.961 |
|  | PCL_R_2_1 | 1.000 | 0.991 | 0.993 |
|  | PCL_L_2_2 | 1.000 | 0.970 | 0.983 |
|  | PCL_R_2_2 | 1.000 | 0.991 | 0.983 |
| Superior temporal gyrus(STG) | STG_L_6_1 | 1.000 | 0.925 | 0.907 |
|  | STG_R_6_1 | 1.000 | 0.991 | 0.993 |
|  | STG_L_6_2 | 1.000 | 0.929 | 0.907 |
|  | STG_R_6_2 | 0.870 | 0.757 | 0.729 |
|  | STG_L_6_3 | 1.000 | 0.924 | 0.907 |
|  | STG_R_6_3 | 1.000 | 0.880 | 0.852 |
|  | STG_L_6_4 | 0.963 | 0.626 | 0.608 |
|  | STG_R_6_4 | 1.000 | 0.991 | 0.993 |
|  | STG_L_6_5 | 0.983 | 0.789 | 0.782 |
|  | STG_R_6_5 | 0.983 | 0.857 | 0.852 |
|  | STG_L_6_6 | 1.000 | 0.783 | 0.782 |
|  | STG_R_6_6 | 1.000 | 0.886 | 0.852 |
| Middle temporal gyrus(MTG) | MTG_L_4_1 | 1.000 | 0.626 | 0.608 |
|  | MTG_R_4_1 | 1.000 | 0.958 | 0.950 |
|  | MTG_L_4_2 | 1.000 | 0.959 | 0.950 |
|  | MTG_R_4_2 | 1.000 | 0.962 | 0.961 |
|  | MTG_L_4_3 | 0.870 | 0.626 | 0.561 |
|  | MTG_R_4_3 | 1.000 | 0.886 | 0.852 |
|  | MTG_L_4_4 | 1.000 | 0.782 | 0.782 |
|  | MTG_R_4_4 | 0.870 | 0.655 | 0.608 |
| Inferior temporal gyrus(ITG) | ITG_L_7_1 | 1.000 | 0.962 | 0.983 |
|  | ITG_R_7_1 | 1.000 | 0.894 | 0.907 |
|  | ITG_L_7_2 | 1.000 | 0.704 | 0.608 |
|  | ITG_R_7_2 | 0.899 | 0.782 | 0.782 |
|  | ITG_L_7_3 | 1.000 | 0.962 | 0.961 |
|  | ITG_R_7_3 | 0.870 | 0.871 | 0.907 |
|  | ITG_L_7_4 | 0.870 | 0.962 | 0.983 |
|  | ITG_R_7_4 | 1.000 | 0.811 | 0.842 |
|  | ITG_L_7_5 | 1.000 | 0.626 | 0.608 |
|  | ITG_R_7_5 | 1.000 | 0.925 | 0.983 |
|  | ITG_L_7_6 | 1.000 | 0.886 | 0.887 |
|  | ITG_R_7_6 | 1.000 | 0.930 | 0.983 |
|  | ITG_L_7_7 | 0.980 | 0.842 | 0.799 |
|  | ITG_R_7_7 | 1.000 | 0.962 | 0.993 |
| Fusiform gyrus(FuG) | FuG_L_3_1 | 1.000 | 0.942 | 0.983 |
|  | FuG_R_3_1 | 1.000 | 0.962 | 0.993 |
|  | FuG_L_3_2 | 1.000 | 0.842 | 0.799 |
|  | FuG_R_3_2 | 1.000 | 0.991 | 0.983 |
|  | FuG_L_3_3 | 0.870 | 0.704 | 0.664 |
|  | FuG_R_3_3 | 1.000 | 0.886 | 0.852 |
| Parahippocampal gyrus(PhG) | PhG_L_6_1 | 1.000 | 0.955 | 0.983 |
|  | PhG_R_6_1 | 1.000 | 0.958 | 0.983 |
|  | PhG_L_6_2 | 1.000 | 0.886 | 0.852 |
|  | PhG_R_6_2 | 1.000 | 0.789 | 0.842 |
|  | PhG_L_6_3 | 0.107 | 0.871 | 0.852 |
|  | PhG_R_6_3 | 1.000 | 0.963 | 0.957 |
|  | PhG_L_6_4 | 1.000 | 0.842 | 0.852 |
|  | PhG_R_6_4 | 0.870 | 0.626 | 0.608 |
|  | PhG_L_6_5 | 1.000 | 0.757 | 0.782 |
|  | PhG_R_6_5 | 1.000 | 0.892 | 0.950 |
|  | PhG_L_6_6 | 0.983 | 0.842 | 0.852 |
|  | PhG_R_6_6 | 1.000 | 0.970 | 0.983 |
| Posterior superior temporal sulcus(pSTS) | pSTS_L_2_1 | 1.000 | 0.871 | 0.852 |
|  | pSTS_R_2_1 | 0.870 | 0.962 | 0.918 |
|  | pSTS_L_2_2 | 0.870 | 0.655 | 0.608 |
|  | pSTS_R_2_2 | 1.000 | 0.842 | 0.782 |
| Superior parietal lobule(SPL) | SPL_L_5_1 | 0.870 | 0.865 | 0.902 |
|  | SPL_R_5_1 | 0.870 | 0.938 | 0.950 |
|  | SPL_L_5_2 | 1.000 | 0.886 | 0.852 |
|  | SPL_R_5_2 | 1.000 | 0.962 | 0.907 |
|  | SPL_L_5_3 | 1.000 | 0.726 | 0.784 |
|  | SPL_R_5_3 | 1.000 | 0.655 | 0.664 |
|  | SPL_L_5_4 | 1.000 | 0.970 | 0.989 |
|  | SPL_R_5_4 | 0.870 | 0.991 | 0.983 |
|  | SPL_L_5_5 | 0.870 | 0.995 | 0.993 |
|  | SPL_R_5_5 | 1.000 | 0.763 | 0.765 |
| Inferior parietal lobule(IPL) | IPL_L_6_1 | 1.000 | 0.721 | 0.700 |
|  | IPL_R_6_1 | 1.000 | 0.842 | 0.813 |
|  | IPL_L_6_2 | 1.000 | 0.924 | 0.907 |
|  | IPL_R_6_2 | 0.870 | 0.626 | 0.631 |
|  | IPL_L_6_3 | 1.000 | 0.783 | 0.784 |
|  | IPL_R_6_3 | 1.000 | 0.626 | 0.608 |
|  | IPL_L_6_4 | 0.870 | 0.845 | 0.907 |
|  | IPL_R_6_4 | 1.000 | 0.626 | 0.608 |
|  | IPL_L_6_5 | 1.000 | 0.757 | 0.782 |
|  | IPL_R_6_5 | 1.000 | 0.704 | 0.782 |
|  | IPL_L_6_6 | 1.000 | 0.991 | 0.983 |
|  | IPL_R_6_6 | 0.870 | 0.991 | 0.993 |
| Precuneus(Pcun) | PCun_L_4_1 | 1.000 | 0.626 | 0.608 |
|  | PCun_R_4_1 | 0.870 | 0.626 | 0.608 |
|  | PCun_L_4_2 | 0.870 | 0.775 | 0.782 |
|  | PCun_R_4_2 | 0.870 | 0.880 | 0.852 |
|  | PCun_L_4_3 | 1.000 | 0.991 | 0.983 |
|  | PCun_R_4_3 | 1.000 | 0.894 | 0.907 |
|  | PCun_L_4_4 | 1.000 | 0.655 | 0.608 |
|  | PCun_R_4_4 | 1.000 | 0.655 | 0.608 |
| Postcentral gyrus(PoG) | PoG_L_4_1 | 1.000 | 0.929 | 0.899 |
|  | PoG_R_4_1 | 1.000 | 0.886 | 0.891 |
|  | PoG_L_4_2 | 1.000 | 0.959 | 0.950 |
|  | PoG_R_4_2 | 1.000 | 0.955 | 0.957 |
|  | PoG_L_4_3 | 0.870 | 0.880 | 0.799 |
|  | PoG_R_4_3 | 0.870 | 0.626 | 0.608 |
|  | PoG_L_4_4 | 0.870 | 0.789 | 0.765 |
|  | PoG_R_4_4 | 0.870 | 0.782 | 0.782 |
| Insular gyrus(INS) | INS_L_6_1 | 0.879 | 0.961 | 0.907 |
|  | INS_R_6_1 | 1.000 | 0.963 | 0.907 |
|  | INS_L_6_2 | 1.000 | 0.991 | 0.989 |
|  | INS_R_6_2 | 1.000 | 0.894 | 0.899 |
|  | INS_L_6_3 | 1.000 | 0.962 | 0.961 |
|  | INS_R_6_3 | 1.000 | 0.884 | 0.873 |
|  | INS_L_6_4 | 1.000 | 0.626 | 0.608 |
|  | INS_R_6_4 | 0.870 | 0.626 | 0.608 |
|  | INS_L_6_5 | 1.000 | 0.991 | 0.961 |
|  | INS_R_6_5 | 1.000 | 0.894 | 0.950 |
|  | INS_L_6_6 | 0.983 | 0.842 | 0.782 |
|  | INS_R_6_6 | 1.000 | 0.991 | 0.950 |
| Cingulate gyrus(CG) | CG_L_7_1 | 1.000 | 0.755 | 0.610 |
|  | CG_R_7_1 | 1.000 | 0.655 | 0.608 |
|  | CG_L_7_2 | 0.870 | 0.757 | 0.782 |
|  | CG_R_7_2 | 1.000 | 0.757 | 0.764 |
|  | CG_L_7_3 | 0.870 | 0.894 | 0.950 |
|  | CG_R_7_3 | 1.000 | 0.842 | 0.842 |
|  | CG_L_7_4 | 1.000 | 0.890 | 0.907 |
|  | CG_R_7_4 | 1.000 | 0.886 | 0.907 |
|  | CG_L_7_5 | 0.870 | 0.782 | 0.729 |
|  | CG_R_7_5 | 0.870 | 0.842 | 0.782 |
|  | CG_L_7_6 | 1.000 | 0.991 | 0.995 |
|  | CG_R_7_6 | 1.000 | 0.962 | 0.993 |
|  | CG_L_7_7 | 1.000 | 0.789 | 0.852 |
|  | CG_R_7_7 | 1.000 | 0.886 | 0.907 |
| MedioVentral occipital cortex(MVOcC) | MVOcC_L_5_1 | 1.000 | 0.789 | 0.852 |
|  | MVOcC_R_5_1 | 0.983 | 0.954 | 0.993 |
|  | MVOcC_L_5_2 | 0.983 | 0.879 | 0.869 |
|  | MVOcC_R_5_2 | 1.000 | 0.962 | 0.978 |
|  | MVOcC_L_5_3 | 1.000 | 0.782 | 0.832 |
|  | MVOcC_R_5_3 | 1.000 | 0.842 | 0.852 |
|  | MVOcC_L_5_4 | 1.000 | 0.959 | 0.989 |
|  | MVOcC_R_5_4 | 1.000 | 0.888 | 0.924 |
|  | MVOcC_L_5_5 | 1.000 | 0.962 | 0.957 |
|  | MVOcC_R_5_5 | 1.000 | 0.955 | 0.950 |
| Lateral occipital cortex(LOcC) | LOcC_L_4_1 | 1.000 | 0.775 | 0.700 |
|  | LOcC_R_4_1 | 1.000 | 0.842 | 0.782 |
|  | LOcC_L_4_2 | 0.870 | 0.757 | 0.631 |
|  | LOcC_R_4_2 | 0.870 | 0.886 | 0.852 |
|  | LOcC_L_4_3 | 0.870 | 0.880 | 0.852 |
|  | LOcC_R_4_3 | 1.000 | 0.949 | 0.907 |
|  | LOcC_L_4_4 | 1.000 | 0.655 | 0.608 |
|  | LOcC_R_4_4 | 1.000 | 0.938 | 0.907 |
|  | LOcC_L_2_1 | 1.000 | 0.892 | 0.872 |
|  | LOcC_R_2_1 | 1.000 | 0.886 | 0.873 |
|  | LOcC_L_2_2 | 0.870 | 0.721 | 0.608 |
|  | LOcC_R_2_2 | 0.983 | 0.653 | 0.608 |
| Amygdala(Amyg) | Amyg_L_2_1 | 1.000 | 0.757 | 0.782 |
|  | Amyg_R_2_1 | 1.000 | 0.929 | 0.907 |
|  | Amyg_L_2_2 | 1.000 | 0.757 | 0.689 |
|  | Amyg_R_2_2 | 1.000 | 0.959 | 0.957 |
| Hippocampus(Hipp) | Hipp_L_2_1 | 1.000 | 0.704 | 0.764 |
|  | Hipp_R_2_1 | 1.000 | 0.962 | 0.984 |
|  | Hipp_L_2_2 | 1.000 | 0.991 | 0.983 |
|  | Hipp_R_2_2 | 1.000 | 0.899 | 0.873 |
| Basal ganglia(BG) | BG_L_6_1 | 1.000 | 0.782 | 0.852 |
|  | BG_R_6_1 | 1.000 | 0.842 | 0.852 |
|  | BG_L_6_2 | 1.000 | 0.757 | 0.782 |
|  | BG_R_6_2 | 0.870 | 0.626 | 0.608 |
|  | BG_L_6_3 | 1.000 | 0.626 | 0.608 |
|  | BG_R_6_3 | 1.000 | 0.991 | 0.997 |
|  | BG_L_6_4 | 1.000 | 0.704 | 0.799 |
|  | BG_R_6_4 | 1.000 | 0.807 | 0.891 |
|  | BG_L_6_5 | 1.000 | 0.845 | 0.852 |
|  | BG_R_6_5 | 0.870 | 0.991 | 0.957 |
|  | BG_L_6_6 | 0.870 | 0.626 | 0.608 |
|  | BG_R_6_6 | 1.000 | 0.704 | 0.782 |
| Thalamus(Tha) | Tha_L_8_1 | 1.000 | 0.826 | 0.782 |
|  | Tha_R_8_1 | 1.000 | 0.755 | 0.608 |
|  | Tha_L_8_2 | 1.000 | 0.782 | 0.765 |
|  | Tha_R_8_2 | 1.000 | 0.655 | 0.664 |
|  | Tha_L_8_3 | 1.000 | 0.842 | 0.782 |
|  | Tha_R_8_3 | 1.000 | 0.783 | 0.782 |
|  | Tha_L_8_4 | 1.000 | 0.782 | 0.782 |
|  | Tha_R_8_4 | 1.000 | 0.626 | 0.608 |
|  | Tha_L_8_5 | 0.870 | 0.783 | 0.782 |
|  | Tha_R_8_5 | 0.870 | 0.782 | 0.782 |
|  | Tha_L_8_6 | 1.000 | 0.892 | 0.852 |
|  | Tha_R_8_6 | 1.000 | 0.886 | 0.867 |
|  | Tha_L_8_7 | 0.870 | 0.970 | 0.950 |
|  | Tha_R_8_7 | 1.000 | 0.886 | 0.852 |
|  | Tha_L_8_8 | 1.000 | 0.704 | 0.764 |
|  | Tha_R_8_8 | 1.000 | 0.626 | 0.608 |
| Cerebellum(CB) | CB_L_I-IV | 1.000 | 0.894 | 0.907 |
|  | CB_R_I-IV | 1.000 | 0.842 | 0.852 |
|  | CB_L_V | 1.000 | 0.886 | 0.924 |
|  | CB_R_V | 1.000 | 0.886 | 0.907 |
|  | CB_L_VI | 1.000 | 0.973 | 0.983 |
|  | CB_V_VI | 0.870 | 0.962 | 0.943 |
|  | CB_R_VI | 1.000 | 0.886 | 0.907 |
|  | CB_L_Crus_I | 1.000 | 0.970 | 0.950 |
|  | CB_R_Crus_I | 0.870 | 0.871 | 0.852 |
|  | CB_L_Crus_II | 1.000 | 0.886 | 0.852 |
|  | CB_V_Crus_II | 1.000 | 0.842 | 0.907 |
|  | CB_R_Crus_II | 1.000 | 0.879 | 0.852 |
|  | CB_L_VIIb | 0.107 | 0.755 | 0.738 |
|  | CB_V_VIIb | 1.000 | 0.929 | 0.957 |
|  | CB_R_VIIb | 1.000 | 0.991 | 0.950 |
|  | CB_L_VIIIa | 0.870 | 0.890 | 0.852 |
|  | CB_V_VIIIa | 1.000 | 0.763 | 0.852 |
|  | CB_R_VIIIa | 1.000 | 0.886 | 0.993 |
|  | CB_L_VIIIb | 1.000 | 0.886 | 0.983 |
|  | CB_V_VIIIb | 1.000 | 0.783 | 0.907 |
|  | CB_R_VIIIb | 1.000 | 0.782 | 0.907 |
|  | CB_L_IX | 1.000 | 0.991 | 0.961 |
|  | CB_V_IX | 1.000 | 0.789 | 0.852 |
|  | CB_R_IX | 1.000 | 0.962 | 0.993 |
|  | CB_L_X | 1.000 | 0.763 | 0.852 |
|  | CB_V_X | 1.000 | 0.871 | 0.907 |
|  | CB_R_X | 1.000 | 0.871 | 0.872 |
| L, left; R, right; V, vermis.  ^a^ All the nodes are from the Human Brainnetome Atlas.  ^b^ The FDR correction was applied to local topological properties. | | | | |

**Table 4**

Changes in nodal topological properties within module 1, 2, 4 and 6 between BL and 6M in relapser

| Region | Node ID ^a^ | *p* value ^b^ | | | |
| --- | --- | --- | --- | --- | --- |
|  |  | Betweenness centrality | Degree centrality | Nodal efficiency | Participation coefficient |
| Superior frontal gyrus(SFG) | SFG_L_7_1 | 0.985 | 0.846 | 0.917 | 0.793 |
|  | SFG_R_7_1 | 0.978 | 0.677 | 0.809 | 0.810 |
|  | SFG_L_7_2 | 0.985 | 0.677 | 0.811 | 0.912 |
|  | SFG_R_7_2 | 0.985 | 0.848 | 0.917 | 0.966 |
|  | SFG_L_7_3 | 0.923 | 0.976 | 0.918 | 0.793 |
|  | SFG_R_7_3 | 0.923 | 0.891 | 0.913 | 0.785 |
|  | SFG_L_7_4 | 0.923 | 0.477 | 0.641 | 0.966 |
|  | SFG_R_7_4 | 0.923 | 0.838 | 0.761 | 0.574 |
|  | SFG_L_7_5 | 0.923 | 0.976 | 0.779 | 0.471 |
|  | SFG_R_7_5 | 0.985 | 0.976 | 0.880 | 0.854 |
|  | SFG_L_7_6 | 0.978 | 0.700 | 0.833 | 0.810 |
|  | SFG_R_7_6 | 0.945 | 0.617 | 0.685 | 0.854 |
|  | SFG_L_7_7 | 0.985 | 0.976 | 0.938 | 0.832 |
|  | SFG_R_7_7 | 0.923 | 0.976 | 0.917 | 0.854 |
| Middle frontal gyrus(MFG) | MFG_L_7_1 | 0.985 | 0.970 | 0.917 | 0.883 |
|  | MFG_R_7_1 | 0.985 | 0.700 | 0.917 | 0.883 |
|  | MFG_L_7_2 | 0.978 | 0.458 | 0.628 | 0.810 |
|  | MFG_R_7_2 | 0.923 | 0.743 | 0.761 | 0.793 |
|  | MFG_L_7_3 | 0.985 | 0.846 | 0.880 | 0.981 |
|  | MFG_R_7_3 | 0.985 | 0.908 | 0.958 | 0.832 |
|  | MFG_L_7_4 | 0.999 | 0.856 | 0.647 | 0.793 |
|  | MFG_R_7_4 | 0.985 | 0.976 | 0.833 | 0.793 |
|  | MFG_L_7_5 | 0.926 | 0.700 | 0.880 | 0.810 |
|  | MFG_R_7_5 | 0.923 | 0.458 | 0.628 | 0.793 |
|  | MFG_L_7_6 | 0.985 | 0.690 | 0.833 | 0.793 |
|  | MFG_L_7_7 | 0.985 | 0.976 | 0.917 | 0.793 |
|  | MFG_R_7_7 | 0.923 | 0.891 | 0.811 | 0.574 |
| Inferior frontal gyrus(IFG) | IFG_L_6_1 | 0.923 | 0.458 | 0.628 | 0.810 |
|  | IFG_R_6_1 | 0.985 | 0.976 | 0.917 | 0.793 |
|  | IFG_L_6_2 | 0.999 | 0.458 | 0.628 | 0.832 |
|  | IFG_R_6_2 | 0.945 | 0.674 | 0.833 | 0.949 |
|  | IFG_L_6_3 | 0.978 | 0.838 | 0.912 | 0.981 |
|  | IFG_R_6_3 | 0.978 | 0.838 | 0.917 | 0.854 |
|  | IFG_L_6_4 | 0.985 | 0.970 | 0.939 | 0.890 |
|  | IFG_R_6_4 | 0.923 | 0.816 | 0.912 | 0.832 |
|  | IFG_L_6_5 | 0.985 | 0.838 | 0.833 | 0.793 |
|  | IFG_R_6_5 | 0.978 | 0.976 | 0.951 | 0.890 |
|  | IFG_L_6_6 | 0.926 | 0.856 | 0.917 | 0.857 |
|  | IFG_R_6_6 | 0.978 | 0.826 | 0.917 | 0.981 |
| Orbital gyrus(OrG) | OrG_L_6_1 | 0.985 | 0.838 | 0.912 | 0.981 |
|  | OrG_R_6_1 | 0.923 | 0.458 | 0.665 | 0.859 |
|  | OrG_L_6_2 | 0.985 | 0.976 | 0.917 | 0.982 |
|  | OrG_R_6_2 | 0.985 | 0.988 | 0.917 | 0.810 |
|  | OrG_L_6_3 | 0.945 | 0.458 | 0.647 | 0.793 |
|  | OrG_R_6_3 | 0.985 | 0.846 | 0.960 | 0.890 |
|  | OrG_L_6_4 | 0.985 | 0.896 | 0.951 | 0.471 |
|  | OrG_R_6_4 | 0.923 | 0.471 | 0.668 | 0.955 |
|  | OrG_L_6_5 | 0.985 | 0.693 | 0.916 | 0.981 |
|  | OrG_R_6_5 | 0.985 | 0.976 | 0.960 | 0.901 |
|  | OrG_L_6_6 | 0.985 | 0.908 | 0.958 | 0.920 |
|  | OrG_R_6_6 | 0.985 | 0.896 | 0.916 | 0.901 |
| Precentral gyrus(PrG) | PrG_L_6_1 | 0.985 | 0.976 | 0.917 | 0.854 |
|  | PrG_R_6_1 | 0.923 | 0.603 | 0.685 | 0.574 |
|  | PrG_L_6_2 | 0.985 | 0.700 | 0.740 | 0.832 |
|  | PrG_R_6_2 | 0.985 | 0.896 | 0.916 | 0.854 |
|  | PrG_L_6_3 | 0.923 | 0.610 | 0.647 | 0.471 |
|  | PrG_R_6_3 | 0.978 | 0.610 | 0.827 | 0.981 |
|  | PrG_L_6_4 | 0.960 | 0.705 | 0.761 | 0.981 |
|  | PrG_R_6_4 | 0.978 | 0.896 | 0.941 | 0.810 |
|  | PrG_L_6_5 | 0.985 | 0.846 | 0.740 | 0.854 |
|  | PrG_R_6_5 | 0.999 | 0.910 | 0.961 | 0.981 |
|  | PrG_L_6_6 | 0.985 | 0.471 | 0.685 | 0.966 |
|  | PrG_R_6_6 | 0.985 | 0.743 | 0.880 | 0.982 |
| Paracentral lobule(PCL) | PCL_L_2_1 | 0.923 | 0.908 | 0.960 | 0.857 |
|  | PCL_R_2_1 | 0.923 | 0.917 | 0.939 | 0.471 |
|  | PCL_L_2_2 | 0.985 | 0.953 | 0.960 | 0.793 |
|  | PCL_R_2_2 | 0.985 | 0.838 | 0.920 | 0.832 |
| Superior temporal gyrus(STG) | STG_L_6_2 | 0.923 | 0.891 | 0.913 | 0.793 |
|  | STG_R_6_2 | 0.923 | 0.705 | 0.758 | 0.785 |
|  | STG_L_6_3 | 0.985 | 0.908 | 0.917 | 0.858 |
|  | STG_R_6_3 | 0.985 | 0.856 | 0.880 | 0.854 |
|  | STG_L_6_4 | 0.923 | 0.458 | 0.628 | 0.912 |
|  | STG_R_6_4 | 0.985 | 0.846 | 0.917 | 0.832 |
|  | STG_L_6_5 | 0.923 | 0.610 | 0.685 | 0.810 |
|  | STG_R_6_5 | 0.999 | 0.846 | 0.880 | 0.912 |
|  | STG_L_6_6 | 0.923 | 0.610 | 0.685 | 0.857 |
|  | STG_R_6_6 | 0.985 | 0.848 | 0.903 | 0.917 |
| Middle temporal gyrus(MTG) | MTG_L_4_1 | 0.923 | 0.458 | 0.641 | 0.793 |
|  | MTG_R_4_1 | 0.985 | 0.908 | 0.917 | 0.949 |
|  | MTG_L_4_2 | 0.978 | 0.846 | 0.918 | 0.471 |
|  | MTG_R_4_2 | 0.985 | 0.793 | 0.917 | 0.966 |
|  | MTG_L_4_3 | 0.923 | 0.098 | 0.154 | 0.854 |
|  | MTG_L_4_4 | 0.944 | 0.603 | 0.740 | 0.917 |
|  | MTG_R_4_4 | 0.923 | 0.477 | 0.685 | 0.854 |
| Inferior temporal gyrus(ITG) | ITG_L_7_4 | 0.923 | 0.924 | 0.951 | 0.890 |
|  | ITG_R_7_4 | 0.985 | 0.617 | 0.833 | 0.890 |
|  | ITG_L_7_6 | 0.985 | 0.846 | 0.917 | 0.917 |
|  | ITG_R_7_6 | 0.985 | 0.954 | 0.958 | 0.810 |
| Posterior superior temporal sulcus(pSTS) | pSTS_L_2_1 | 0.985 | 0.700 | 0.833 | 0.966 |
|  | pSTS_R_2_1 | 0.923 | 0.910 | 0.917 | 0.901 |
|  | pSTS_L_2_2 | 0.923 | 0.603 | 0.641 | 0.981 |
|  | pSTS_R_2_2 | 0.985 | 0.793 | 0.685 | 0.965 |
| Superior parietal lobule(SPL) | SPL_R_5_3 | 0.999 | 0.458 | 0.641 | 0.854 |
|  | SPL_L_5_4 | 0.923 | 0.976 | 0.917 | 0.859 |
|  | SPL_R_5_4 | 0.923 | 0.976 | 0.939 | 0.883 |
| Inferior parietal lobule(IPL) | IPL_L_6_2 | 0.985 | 0.908 | 0.872 | 0.857 |
|  | IPL_R_6_2 | 0.923 | 0.664 | 0.779 | 0.883 |
|  | IPL_L_6_3 | 0.945 | 0.458 | 0.641 | 0.810 |
|  | IPL_L_6_4 | 0.923 | 0.700 | 0.951 | 1.000 |
|  | IPL_R_6_4 | 0.985 | 0.477 | 0.647 | 0.890 |
|  | IPL_L_6_5 | 0.970 | 0.587 | 0.804 | 0.912 |
|  | IPL_R_6_5 | 0.923 | 0.458 | 0.685 | 0.912 |
|  | IPL_L_6_6 | 0.944 | 0.865 | 0.917 | 0.912 |
|  | IPL_R_6_6 | 0.923 | 0.846 | 0.917 | 0.981 |
| Precuneus(Pcun) | PCun_L_4_4 | 0.985 | 0.458 | 0.628 | 0.912 |
|  | PCun_R_4_4 | 0.985 | 0.458 | 0.641 | 0.966 |
| Postcentral gyrus(PoG) | PoG_L_4_1 | 0.999 | 0.976 | 0.917 | 0.858 |
|  | PoG_R_4_1 | 0.923 | 0.705 | 0.833 | 0.854 |
|  | PoG_L_4_2 | 0.926 | 0.896 | 0.917 | 0.857 |
|  | PoG_R_4_2 | 0.985 | 0.953 | 0.917 | 0.955 |
|  | PoG_L_4_3 | 0.923 | 0.856 | 0.685 | 0.917 |
|  | PoG_R_4_3 | 0.923 | 0.471 | 0.647 | 0.981 |
|  | PoG_L_4_4 | 0.978 | 0.603 | 0.685 | 0.471 |
|  | PoG_R_4_4 | 0.923 | 0.976 | 0.917 | 0.900 |
| Insular gyrus(INS) | INS_L_6_1 | 0.923 | 0.976 | 0.917 | 0.854 |
|  | INS_R_6_1 | 0.985 | 0.793 | 0.761 | 0.793 |
|  | INS_L_6_2 | 0.978 | 0.856 | 0.918 | 0.681 |
|  | INS_R_6_2 | 0.967 | 0.826 | 0.917 | 0.574 |
|  | INS_L_6_3 | 0.999 | 0.884 | 0.917 | 0.595 |
|  | INS_R_6_3 | 0.923 | 0.896 | 0.917 | 0.471 |
|  | INS_L_6_4 | 0.978 | 0.846 | 0.903 | 0.793 |
|  | INS_R_6_4 | 0.985 | 0.705 | 0.668 | 0.471 |
|  | INS_L_6_5 | 0.985 | 0.970 | 0.833 | 0.810 |
|  | INS_R_6_5 | 0.978 | 0.846 | 0.939 | 0.854 |
|  | INS_L_6_6 | 0.923 | 0.721 | 0.647 | 0.471 |
|  | INS_R_6_6 | 0.944 | 0.976 | 0.903 | 0.901 |
| Cingulate gyrus(CG) | CG_L_7_1 | 0.985 | 0.471 | 0.628 | 0.966 |
|  | CG_R_7_1 | 0.923 | 0.458 | 0.628 | 0.981 |
|  | CG_L_7_2 | 0.985 | 0.603 | 0.685 | 0.574 |
|  | CG_R_7_2 | 0.923 | 0.477 | 0.641 | 0.471 |
|  | CG_L_7_3 | 0.923 | 0.856 | 0.917 | 0.911 |
|  | CG_R_7_3 | 0.978 | 0.856 | 0.833 | 0.605 |
|  | CG_L_7_5 | 0.923 | 0.910 | 0.758 | 0.793 |
|  | CG_R_7_5 | 0.923 | 0.891 | 0.686 | 0.810 |
|  | CG_L_7_6 | 0.999 | 0.896 | 0.974 | 0.810 |
|  | CG_R_7_6 | 0.926 | 0.891 | 0.958 | 0.793 |
|  | CG_L_7_7 | 0.985 | 0.846 | 0.913 | 0.793 |
|  | CG_R_7_7 | 0.985 | 0.816 | 0.833 | 0.981 |
| Amygdala(Amyg) | Amyg_L_2_1 | 0.923 | 0.458 | 0.641 | 0.574 |
|  | Amyg_R_2_1 | 0.985 | 0.610 | 0.647 | 0.785 |
|  | Amyg_L_2_2 | 0.985 | 0.458 | 0.641 | 0.850 |
|  | Amyg_R_2_2 | 0.999 | 0.816 | 0.917 | 0.793 |
| Basal ganglia(BG) | BG_L_6_1 | 0.985 | 0.610 | 0.880 | 0.927 |
|  | BG_R_6_1 | 0.985 | 0.700 | 0.833 | 0.890 |
|  | BG_L_6_2 | 0.923 | 0.846 | 0.833 | 0.793 |
|  | BG_R_6_2 | 0.923 | 0.471 | 0.641 | 0.793 |
|  | BG_L_6_3 | 0.923 | 0.471 | 0.641 | 0.810 |
|  | BG_R_6_3 | 0.985 | 0.949 | 0.958 | 0.966 |
|  | BG_L_6_4 | 0.960 | 0.610 | 0.880 | 0.471 |
|  | BG_R_6_4 | 0.923 | 0.970 | 0.951 | 0.920 |
|  | BG_L_6_5 | 0.999 | 0.838 | 0.880 | 0.785 |
|  | BG_R_6_5 | 0.923 | 0.926 | 0.951 | 0.832 |
|  | BG_L_6_6 | 0.923 | 0.477 | 0.685 | 0.678 |
|  | BG_R_6_6 | 0.985 | 0.561 | 0.833 | 0.810 |
| Thalamus(Tha) | Tha_L_8_1 | 0.985 | 0.610 | 0.761 | 0.901 |
|  | Tha_R_8_1 | 0.999 | 0.471 | 0.641 | 0.793 |
|  | Tha_L_8_2 | 0.999 | 0.908 | 0.811 | 0.574 |
|  | Tha_R_8_2 | 0.985 | 0.477 | 0.641 | 0.793 |
|  | Tha_L_8_3 | 0.999 | 0.793 | 0.779 | 0.854 |
|  | Tha_R_8_3 | 0.985 | 0.836 | 0.811 | 0.810 |
|  | Tha_L_8_4 | 0.978 | 0.610 | 0.641 | 0.859 |
|  | Tha_R_8_4 | 0.923 | 0.603 | 0.628 | 0.574 |
|  | Tha_L_8_5 | 0.923 | 0.677 | 0.761 | 0.854 |
|  | Tha_R_8_5 | 0.923 | 0.985 | 0.917 | 0.793 |
|  | Tha_L_8_6 | 0.985 | 0.976 | 0.880 | 0.785 |
|  | Tha_R_8_6 | 0.923 | 0.976 | 0.917 | 0.981 |
|  | Tha_L_8_7 | 0.923 | 0.976 | 0.918 | 0.901 |
|  | Tha_R_8_7 | 0.985 | 0.848 | 0.917 | 0.981 |
|  | Tha_L_8_8 | 0.985 | 0.477 | 0.647 | 0.793 |
|  | Tha_R_8_8 | 0.985 | 0.458 | 0.628 | 0.574 |
| Cerebellum(CB) | CB_L_I-IV | 0.985 | 0.838 | 0.917 | 0.920 |
|  | CB_R_I-IV | 0.978 | 0.700 | 0.917 | 0.982 |
|  | CB_L_V | 0.923 | 0.846 | 0.917 | 0.793 |
|  | CB_R_V | 0.985 | 0.846 | 0.917 | 0.901 |
|  | CB_L_VI | 0.923 | 0.896 | 0.958 | 0.890 |
|  | CB_V_VI | 0.985 | 0.903 | 0.917 | 0.810 |
|  | CB_R_VI | 0.923 | 0.953 | 0.917 | 0.858 |
|  | CB_L_Crus_I | 0.985 | 0.976 | 0.917 | 0.981 |
|  | CB_R_Crus_I | 0.985 | 0.846 | 0.917 | 0.981 |
|  | CB_L_Crus_II | 0.978 | 0.976 | 0.917 | 0.793 |
|  | CB_V_Crus_II | 0.985 | 0.471 | 0.833 | 0.981 |
|  | CB_R_Crus_II | 0.985 | 0.677 | 0.740 | 0.793 |
|  | CB_L_VIIb | 0.999 | 0.838 | 0.740 | 0.793 |
|  | CB_V_VIIb | 0.985 | 0.477 | 0.833 | 0.966 |
|  | CB_R_VIIb | 0.985 | 0.953 | 0.917 | 0.785 |
|  | CB_L_VIIIa | 0.926 | 0.976 | 0.917 | 0.858 |
|  | CB_V_VIIIa | 0.985 | 0.458 | 0.708 | 0.854 |
|  | CB_R_VIIIa | 0.985 | 0.603 | 0.917 | 0.854 |
|  | CB_L_VIIIb | 0.923 | 0.610 | 0.958 | 0.911 |
|  | CB_V_VIIIb | 0.923 | 0.477 | 0.811 | 0.901 |
|  | CB_R_VIIIb | 0.985 | 0.477 | 0.951 | 0.941 |
|  | CB_L_IX | 0.999 | 0.846 | 0.966 | 0.890 |
|  | CB_V_IX | 0.985 | 0.700 | 0.833 | 0.858 |
|  | CB_R_IX | 0.978 | 0.884 | 0.958 | 0.917 |
|  | CB_L_X | 0.927 | 0.477 | 0.811 | 0.883 |
|  | CB_V_X | 0.985 | 0.610 | 0.833 | 0.793 |
|  | CB_R_X | 0.923 | 0.471 | 0.779 | 0.981 |
| L, left; R, right; V, vermis.  ^a^ All the nodes are from the Human Brainnetome Atlas.  ^b^ The FDR correction was applied to local topological properties. | | | | | |

**Table 5**

Comparison of changes in global network metrics between responder and relapser

| Global metric | Responder (Δ, mean ± SD) | Relapser (Δ, mean ± SD) | *p* value |
| --- | --- | --- | --- |
| σ | 0.0476±0.0771 | 0.0292±0.1308 | 0.806 |
| Lp | -0.0067±0.0105 | -0.0001±0.0235 | 0.514 |
| λ | -0.0022±0.0045 | 0.0007±0.0098 | 0.435 |
| Cp | -0.0040±0.0098 | -0.0011±0.0180 | 0.662 |
| γ | 0.0519±0.0940 | 0.0319±0.1343 | 1.000 |
| Eloc | -0.0007±0.0058 | 0.0000±0.0078 | 0.817 |
| Eg | 0.0016±0.0023 | 0.0003±0.0054 | 0.414 |
| Q | 0.0002±0.0117 | 0.0047±0.0205 | 0.562 |
| Changes were calculated as 6-month follow-up minus baseline and are presented as mean ± SD. | | | |
